# Anatase–Gentamicin Electrophoretic Coatings on Polyurethane‐Modified Titanium: Antibacterial Performance, Biocompatibility, and Drug Delivery

**DOI:** 10.1155/ijbm/7643259

**Published:** 2026-04-28

**Authors:** Fabiola A. Gutiérrez-Mejía, Rossana F. Vargas-Coronado, Claudia Vásquez-López, Luis Díaz-Ballote, Claribel Huchin-Chan, Fabiola E. Villa-de-la-Torre, Victor E. Arana-Argaez, Raúl Rosales-Ibáñez, Arely M. González-González, Raymundo Cruz-Pérez, Juan V. Cauich-Rodríguez

**Affiliations:** ^1^ Materials Department, Centro de Investigación Científica de Yucatán (CICY), Mérida, Yucatán, Mexico, cicy.mx; ^2^ Physics Department, Universidad de Sonora (UniSon), Hermosillo, Sonora, Mexico; ^3^ Applied Physics Department, Centro de Investigación y Estudios Avanzados del IPN Unidad Mérida (CINVESTAV), Mérida, Yucatán, Mexico; ^4^ Chemistry Faculty, Universidad Autónoma de Yucatán (UADY), Mérida, Yucatán, Mexico; ^5^ Laboratory of Tissue Engineering and Translational Medicine, Faculty of Higher Education Iztacala, National Autonomous University of Mexico (UNAM), México City, Mexico, unam.mx; ^6^ Research Laboratory in Nano and Dental Biomaterials, Faculty of Higher Studies Iztacala, National Autonomous University of Mexico (UNAM), México City, Mexico, unam.mx; ^7^ Department of Infectomics and Molecular Pathogenesis, Center for Research and Advanced Studies (CINVESTAV), México City, Mexico, cinvestav.mx

**Keywords:** anatase, biocompatibility, electrophoretic deposition, gentamicin, surface coatings

## Abstract

Infections, limited biocompatibility, and material degradation remain major challenges for metallic implants in biomedical applications. To address these issues, this study presents a multifunctional coating strategy for Grade 2 titanium using a base layer of segmented polyurethane (Tecoflex SG‐80A), followed by the co‐deposition of titanium dioxide nanoparticles and gentamicin sulfate. Corrosion polarization tests revealed enhanced passivation of polyurethane‐coated surfaces with no signs of pitting corrosion. Coatings showed porous microstructures with both nanoparticles and antibiotics distributed within and along pore edges. Energy‐dispersive X‐ray spectroscopy (EDX) confirmed the surface presence of both components. Thermogravimetric analysis indicated loadings of 0.17 ± 0.02 mg of gentamicin and 0.30 ± 0.04 mg of TiO_2_ per specimen. SEM and AFM analyses showed that over 86% of the surface was covered with gentamicin and nanoparticles. Contact angle measurements revealed a hydrophilic character (35°) for coatings containing both gentamicin and TiO_2_ nanoparticles, favorable for biological interactions. Cytotoxicity assays using dental pulp mesenchymal cells and fibroblasts demonstrated no cytotoxic effects after 72 h, whereas antibacterial tests against *Staphylococcus aureus* and *Escherichia coli* indicated inhibitory effects. Gentamicin release from the coatings followed the Korsmeyer–Peppas model, suggesting a diffusion‐driven profile. These results support the development of durable, biocompatible, and antibacterial coatings for titanium implants that can reduce infection risk, enhance corrosion resistance, and support tissue integration.

## 1. Introduction

Titanium and its alloys are extensively used in medical implants due to their exceptional corrosion resistance, bioinertness, and favorable mechanical properties [1, 2]. These alloys are vital components in orthopedic implants, dental prosthetics, and cardiovascular devices, valued for their mechanical strength, biocompatibility, durability, and proven osseointegration capabilities [3–5].

Despite these significant advantages, titanium implants still present challenges, including potential toxic effects arising from chemical reactions with body fluids, suboptimal tissue and bone responses in some instances, and inherent lack of antibacterial properties [6–8]. This underscores an urgent need for multifunctional surface modifications to equip titanium with the enhanced properties required for successful long‐term application in the biomedical field.

Surface treatments and coatings on titanium implants have gained significant attention for optimizing their interactions with the biological host environment. Various surface modification techniques have been explored, including additive manufacturing (AM), which offers cost‐effective and sustainable coating application [9]. Physical vapor deposition (PVD) methods have been leveraged to create multilayer coatings on Ti‐based alloys and stainless steel (SS), enhancing wear resistance and reducing friction coefficients [10]. Mechanical treatments like grit blasting alter surface topography to promote biological interactions [11]. Furthermore, chemical modifications include hydrogen peroxide treatment and acidic etching treatment [12].

In particular, a well‐established technique for metallic surface coating is electrophoretic deposition (EPD) [13]. EPD is a versatile technique known for its scalability in coating complex shapes with homogeneous thickness and for requiring a facile and cost‐effective setup. Unlike PVD, which is often limited by “line‐of‐sight” constraints and high energy consumption, EPD operates under ambient conditions and utilizes electric fields to achieve uniform coverage on highly complex or porous geometries. EPD also enables the co‐deposition of polymers and nanoparticles (NPs), overcoming the non‐uniformity inherent in passive methods like dip‐coating [14–16]. This process ensures a dense, homogeneous integration of multicomponent matrices, offering a scalable and cost‐effective alternative to the aforementioned surface treatments for antimicrobial biocoatings.

Overall, EPD provides a powerful and adaptable strategy for fabricating multifunctional antimicrobial coatings that integrate NPs, ceramics, and antibiotics to prevent implant‐related infections while enhancing the interfacial biocompatibility of the metallic substrate. In particular, antibiotics such as gentamicin and ciprofloxacin have been successfully incorporated into hydroxyapatite (HAP)–polymer composite coatings and deposited using the EPD technique. For instance, HAP NPs loaded with antibiotics (gentamicin and ciprofloxacin) were synthesized and coated by EPD on titanium, where the obtained coatings exhibited prolonged drug release and antibacterial activity [17]. Similarly, other EPD‐based systems have incorporated gentamicin into HAP and chitosan, yielding hybrid coatings that promote both antibacterial and bioactive behavior [18, 19]. More recently, chitosan/nano‐hydroxyapatite/silver nanoparticle (CS/nanoHAp/AgNP) coatings produced via EPD have shown tunable adhesion, bioactivity, and controlled silver release, providing a balance between antibacterial efficacy and coating stability on titanium substrates. Likewise, in situ EPD of tannic acid‐loaded zein particles combined with copper‐doped bioactive glass has yielded multifunctional coatings capable of sustained drug release and improved adhesion [20]. Other coatings typically combine bioactive materials like chitosan, bioactive glass, or silk fibroin with antibiotics such as gentamicin to enhance osseointegration and prevent infections.

Polyurethane (PU) coatings on titanium substrates have also been explored for various biomedical applications. PU‐based coatings can enhance biocompatibility and reduce thrombosis in cardiovascular devices [21]. Porous PU layers created using enhanced solvent casting/particulate leaching methods can improve endothelial cell adhesion [22]. Furthermore, bioactivity and bone regeneration can be enhanced by incorporating HAP NPs and magnesium particles into PU coatings applied to alkali‐treated titanium surfaces [23]. In addition, multifunctional PU‐based composite coatings that combine graphene—due to its antibacterial properties—and β‐tricalcium phosphate (β‐TCP)—for its osteoconductive capabilities—have shown potential in bone surgery and dental applications [24].

In this work, we propose a multifunctional coating consisting of commercial PU, TiO_2_ NPs, and gentamicin using titanium as a substrate. Incorporating a segmented polyurethane (SPU) layer into EPD‐based systems presents an innovative strategy to further enhance coating performance. The rationale behind using a layer of SPU on titanium is based not only on the expected improved corrosion resistance but also on the SPU’s tunable polymeric structure, which enables it to function as a drug reservoir or bioactive carrier, facilitating controlled antibiotic release while maintaining surface bioactivity. TiO_2_ NPs were used to provide a bioactive substrate for improved osteointegration, while gentamicin was used as a model antibacterial in contrast to silver or chitosan commonly used in EPD. We evaluate the performance of the coatings based on their bioactivity and physicochemical properties.

## 2. Materials and Methods

Titanium dioxide NPs of 21 nm, chloroform, acetic acid, and gentamicin sulfate (G) were purchased from Sigma‐Aldrich (MO, USA). PU Tecoflex SG‐80A (SPU) was supplied by Lubrizol (Ohio, USA), and Grade 2 titanium plates (15 cm × 15 cm × 0.15 cm) were obtained from Jindong Store (Beijing, China). Metal coupons were cut into coin‐shaped discs with a diameter of 14 mm, each featuring a small tab to allow electrical connection. The surfaces were mechanically polished using 220‐grit sandpaper, followed by a finer polish with 600‐grit paper. Atomic force microscopy (AFM) measurements revealed an average surface roughness of Rq = 0.53 ± 0.21 μm for this metal plate.

### 2.1. Surface Preparation and Deposition Method

PU‐coated titanium (Ti) coupons were prepared by dissolving Tecoflex SG‐80A in chloroform at a concentration of 50 mg/mL. The Ti substrates were immersed in the SPU solution for 1 s, then air‐dried at 25°C for 48 h and stored under ambient dry conditions until further use. Dispersions of TiO_2_ NPs were prepared in deionized water at a concentration of 1.25 mg/mL. The pH of the suspension was adjusted to 3 using 4% v/v acetic acid to ensure a positive zeta potential of the TiO_2_ NP, thereby promoting their migration toward the cathode during EPD. Suspensions with antibiotics were prepared by adding 1 mg/mL to a previously prepared NP suspension. EPD was also performed for samples with only gentamicin; for this, 1 mg/mL of G solution was dissolved in water at pH adjusted with acetic acid. For homogenization, suspensions were sonicated for 30 min and stirred. Fresh solutions were used for each deposition. The weight and thickness of the coupons were measured before and 48 h after deposition with an Ohaus digital balance and a Mitutoyo 547‐500S digital thickness gauge with flat anvil.

A direct voltage source (MPS‐6005L‐1, MATRIX, China) was used to produce the electrophoretic deposits. The counter electrode was composed of a bare 316L SS plate acting as the anode and a Tecoflex‐coated Ti round coupon performing as the cathode. The separation distance between plates was fixed to 8 mm, consistent with the range commonly reported for EPD, with a maximum separation of 1 cm [25]. Deposition was carried out by immersing the electrodes in the NP suspensions under an applied voltage of 25 V for 3 min. Following deposition, the specimens were dried at 25°C for a minimum of 48 h before subsequent analysis.

These parameters were selected based on cited literature [18, 19] and the following considerations. An applied voltage of 25 V was chosen because lower voltages did not result in sufficient coating coverage, whereas higher voltages (30–50 V) were associated with deterioration of the polymer layer. Consequently, an intermediate voltage was selected to balance deposition efficiency and coating integrity. The deposition time was determined from preliminary experiments on samples containing NP and G; extended deposition times led to observable changes in the suspension color (from white to yellow) after approximately 10 min, which may indicate the occurrence of undesired physicochemical changes. To reduce the likelihood of such effects, a shorter deposition time was selected.

### 2.2. Polarization Curves

Electrochemical measurements were performed in a Gamry reference 600 series potentiostat (Gamry Instruments, Inc., USA) in a conventional three‐electrode cell setup according to the standard ASTM G61‐86(2018) [26], which applies for Fe‐, Ni‐, and Co‐based alloys but with screening parameters (scan rate and reverse potential) consistent for titanium‐based medical devices as outlined in ASTM F2129 [27], with electrochemical parameters specifically adjusted to be technically equivalent to those prescribed in ASTM F2129 for titanium‐based medical devices. A platinum electrode from StonyLab, United States, was used as the auxiliary electrode, while an Ag/AgCl electrode was the reference electrode, as previously described [28]. Working electrodes comprised bare Ti and SPU‐coated Ti coupons, each with a diameter of 14 mm and a surface area of 1.54 cm^2^. Therefore, measurements were performed on an aqueous solution of deoxygenated, deionized water with 3.5% NaCl by weight. All Ti disks were prepared by grinding with 240‐grit paper and polishing with 600‐grit SiC paper, followed by ethanol cleaning and rinsing with deionized water. Following this treatment, some samples were characterized as bare substrates, while others were coated and characterized thereafter. To stabilize the surface at the open circuit potential (EOCP), each specimen was immersed in the electrolyte for 1 h before measurements. Cyclic polarization curves were then recorded starting at −0.15 V (vs. Ag/AgCl) relative to the EOCP. A scan rate of 0.167 mV s^−1^, consistent with ASTM F2129 recommendations, was employed. The scan direction was reversed at a vertex potential of 0.65 V (vs. Ag/AgCl). The EOCP stabilized at ≈ −212 mV for bare Ti and at −223 mV for coated Ti with SPU. Results were analyzed with the Echem Analyst software (Gamry Instruments, Inc., USA) to obtain characteristic parameters of the polarization curves.

### 2.3. Physicochemical Characterization

Zeta potential measurements of the TiO_2_ suspensions (≈1 mg/mL) dispersed in deionized water at various pH values were carried out using a NANO ZEN3600 instrument (Malvern, Worcestershire, UK). The pH of the suspensions was adjusted using 0.1 M NaOH and 0.1 M HCl to obtain a range of acidic and basic conditions. Each reported zeta potential value corresponds to the average of three independent measurements (*n* = 3).

Fourier‐transform infrared (FTIR) spectra were acquired using a Thermo Scientific Nicolet 8700 spectrometer (MA, USA) equipped with an attenuated total reflectance (ATR) accessory, operating over the 4000–650 cm^−1^ spectral range, averaging 100 scans at a resolution of 4 cm^−1^. Raman spectra were collected using an inVia Renishaw Raman spectrometer coupled with a 633 nm argon laser as the excitation radiation source (IL, USA). The system measured the Raman shift between 3200 and 100 cm^−1^. Some of the methods used in this section are also employed in [29], and wording is partially similar for some of the techniques described.

### 2.4. Structure, Morphology and Quantification of Deposits

Morphology and phase changes were evaluated using AFM with a Bruker Innova IRIS (MA, USA) operating in tapping mode. AFM images were processed using Gwyddion open‐source software to extract surface characteristics such as root mean square roughness (Rq) and topographical features. Phase contrast images from AFM were further processed with ImageJ software using the “Analyze Particles” function to estimate surface coverage.

Scanning electron microscopy (SEM) was performed using either a JSM‐6360LV or a JSM‐7601F microscope (JEOL, Tokyo, Japan) at an accelerating voltage of 20 kV to examine surface morphology. Elemental mapping and compositional analysis were carried out using energy‐dispersive X‐ray spectroscopy (EDX) with an INCA X‐sight Model 7582 system (Oxford Instruments). Specimens were dried at room temperature, cut to appropriate size, and mounted on aluminum stubs using conductive carbon tape. To prevent charging, a thin conductive layer of gold was sputter‐coated on the surface.

The surface density and mass fraction of the coatings were estimated using thermogravimetric analysis (TGA). Measurements were performed with a TGA‐7 instrument (Perkin‐Elmer, CT, USA). To ensure compatibility with the instrument’s specimen mass requirements, the coatings were carefully peeled from the metal coupons using a clean and sharp cutter and tweezers. Specimens (three replicates) were weighed prior to analysis with the equipment scales. Specimens of known mass were then heated from 50°C to 700°C at a rate of 10°C/min under a nitrogen atmosphere. From the initial (m_0_) and residual (m_f_) masses of SPU, NP, and gentamicin, measured separately and combined, a weight percentage (m_f_/m_0_) was obtained. Then the mass fraction of NP and gentamicin was estimated in EPD specimens as well as the surface density. Using the mass loss of the individual SPU, G, and NP and combinations of SPU + G and SPU + TiO_2_ prepared by EPD, the individual masses of the species were obtained.

Contact angle (CA) measurements were performed using a Ramé‐Hart goniometer/tensiometer equipped with DropImage Advanced v2.8 software. The static water CA was determined using the sessile drop method with deionized water at room temperature. Measurements were conducted on three independent specimens, and the CA was recorded at 10‐s intervals 5 times following droplet deposition. Three different droplets for each coating were analyzed.

### 2.5. Antimicrobial Susceptibility Assay

To assess bacterial susceptibility, *Staphylococcus aureus* (*S. aureus*) (ATCC 25923) and a wild‐type strain of *Escherichia coli* (*E. coli*) were used. The initial bacterial concentration was adjusted by measuring the optical density (OD) at 600 nm. All culture medium was autoclaved; the process was conducted at 121°C under a pressure of 1.5 bar for 20 min. Cultures were grown in Mueller–Hinton broth (MHB) and standardized to 0.5 on the McFarland scale, equivalent to approximately 1 × 10^8^ CFU/mL. A volume of 1.7 mL of the bacterial suspension was then mixed with 168.3 mL of Mueller–Hinton agar to obtain a 1:100 dilution. The coated samples were sterilized using the ultraviolet (UVC) unit integrated within a laminar flow fume hood. The samples were positioned below the light source and exposed to UV radiation *λ* = 254 nm for 30 min per side.

Sterilized specimens were placed in 150 mm petri dishes and covered with about 55 mL of the inoculated agar. At least three plates were incubated at 37°C for 24 h, after which the diameter of the inhibition zones was measured to evaluate antibacterial activity.

As a reference for the antimicrobial susceptibility assay, specimens were prepared by simple adsorption of gentamicin onto SPU‐coated Ti coupons (SPU + G_ref_). For this, gentamicin was dispersed in water, and the pH was adjusted to 3 using acetic acid. A total of 100 μL of a 1 mg/mL gentamicin suspension (equivalent to 100 μg) was applied onto each SPU‐coated coupon (in triplicate) and left to dry at room temperature for 48 h.

### 2.6. Antimicrobial Activity by the Microdilution Method

A microdilution technique was used to quantify the in vitro antimicrobial activity of material extracts. This complementary method was used only with *S*. *aureus* suspensions in the presence of the studied materials, which were prediluted in MHB. The procedure consisted of four main steps: preparation of material extracts, bacterial inoculation, incubation for microbial growth, and measurement of bacterial survival. To prepare the extracts, Ti coupons were sterilized in a laminar flow cabinet via three 30‐min UV irradiation cycles and incubated in 2 mL of MHB for 24 h. A bacterial inoculum was prepared from a fresh culture in a test tube containing 5 mL of MHB and incubated at 37°C for 24 h. After incubation, the bacterial suspension was centrifuged at 4000 rpm for 15 min in Eppendorf tubes to isolate the bacterial pellet. The pellet was washed three times with PBS, and the absorbance of the bacterial inoculum was adjusted to 0.1 OD at 600 nm. This inoculum was then diluted 10‐fold in MHB. For bacterial exposure, inoculates were prepared by mixing 30 μL of the bacterial solution with 270 μL of the material extracts. The solutions were incubated for 24 h at 37°C. After incubation, bacterial survival was assessed using a 96‐well microplate reader (Cytation 3 Biotek), and measurements of the OD were taken at 600 nm. All treatments were tested in triplicate (*n* = 3).

Non‐inoculated MHB served as a negative control (sterility control) to establish a baseline for optical measurements and to ensure the absence of environmental contamination throughout the experimental procedure. Conversely, broth inoculated with the bacterial culture in the absence of any antimicrobial agents was utilized as a positive control (growth control). This control (Figure 1(b)) validates the nutritional suitability of the medium and provides a standardized reference for normal microbial proliferation, ensuring that any observed inhibition in the test samples can be accurately attributed to the experimental coatings.

FIGURE 1Antibacterial response of EPD coatings against (a) *S. aureus* and *E. coli* inhibition halo and (b) antimicrobial activity in liquid culture medium on *S. aureus*. Data are presented as mean ± SD (*n* = 3). Statistical analysis was performed using one‐way ANOVA followed by Tukey’s post hoc test. *p* < 0.05 was considered significant.

**Figure:**
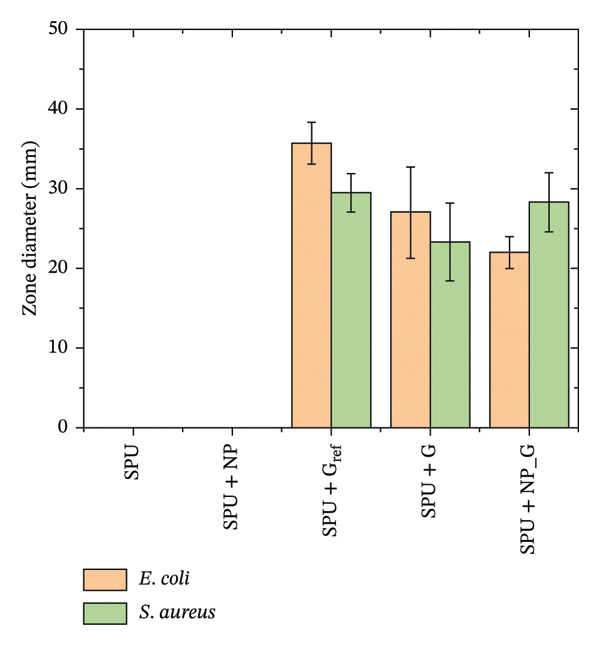
(a)

**Figure:**
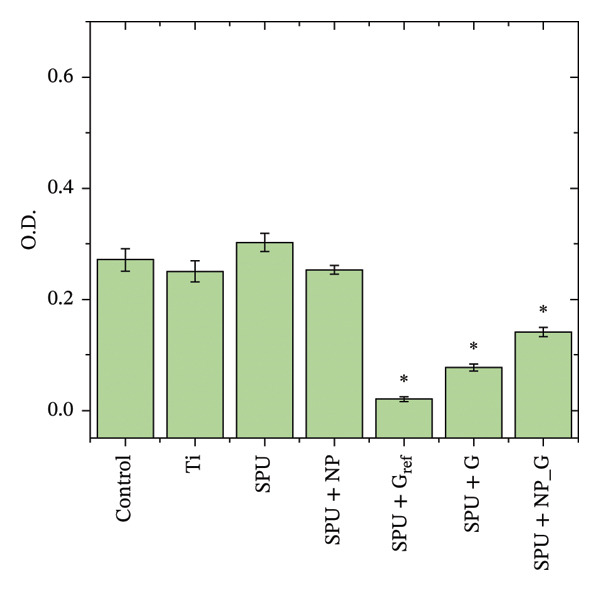
(b)

### 2.7. Cell Viability Assays

Cytotoxicity was evaluated using human fibroblasts (Detroit 548, ATCC CCL‐116^TM^, RRID:CVCL_2432 from the Cellosaurus database; American Type Culture Collection, Rockville, MD, USA) following an indirect extract‐based assay. Material extracts were obtained by incubating coated disks in low‐glucose Dulbecco’s Modified Eagle’s Medium (DMEM; Caisson Laboratories, Smithfield, UT) supplemented with 10% fetal calf serum (Biowest) and 1% penicillin–streptomycin (Sigma‐Aldrich). The incubation was carried out in sterile 48‐well plates at 37°C in a humidified atmosphere containing 5% CO_2_, using a ratio of one disk per 1.5 mL of culture medium. Samples were sterilized in a laminar flow cabinet via three 30‐min UV irradiation cycles. Surfaces were turned between cycles to ensure uniform exposure.

For the cytotoxicity assay, fibroblasts were seeded in 96‐well plates at a density of 1 × 10^4^ cells/well in 100 μL of supplemented DMEM. After 24 h of cell adhesion, the culture medium was replaced with the respective material extracts, and cells were incubated for 24, 48, and 72 h. Cell viability was assessed using the Resazurin reduction assay, and absorbance was measured at 570 nm and 600 nm using a Cytation 3 plate reader (BioTek). Results were expressed as percentage viability relative to untreated control cells.

The viability % was calculated according to the following equation:

(1) % v=O2−A1−O1−A2O2P1−O1P2,

where *O*_1_ and *O*_2_ represent the molar extinction coefficients of oxidized resazurin (blue) at 570 nm (*ε* = 80,586) and 600 nm (*ε* = 117,216), respectively. *A*_1_ and *A*_2_ correspond to the absorbance values of the test wells at 570 nm and 600 nm, while *P*_1_ and *P*_2_ represent the absorbance of the positive growth control wells (cells with resazurin and without test specimen) at 570 nm and 600 nm, respectively [30]. Cell viability (%) was calculated based on these values, and the results were expressed as the mean ± standard deviation from four independent replicates.

Alternatively, the cytotoxicity on dental pulp mesenchymal stem cells (DPMSCs) derived from Swine was evaluated. Animal care and use were approved by the ethical committee (CE/FESI/052017/1174). The isolation, culture, and phenotyping of the primary cells are reported in [31]. For this, an Alamar Blue assay (TermoFisher, Pittsburgh, USA) was used following ISO 10993‐5, which outlines the in vitro evaluation of cytotoxicity. DPMSCs (1 × 10^3^) were seeded in triplicate onto the scaffolds or directly onto plastic surfaces as controls in a 96‐well culture dish (TPP, Trasadingen). Cells were cultured in low‐glucose Dulbecco’s Modified Eagle Medium (DMEM; Gibco, Merelbeke, Belgium), supplemented with 10% fetal bovine serum (FBS; Gibco) and 1% antibiotic–antimycotic solution (PAA, The Cell Culture Company). Cultures were maintained under standard conditions at 37°C and 5% CO_2_.

At 24, 48, and 72 h and 7 days, the medium was removed, and 90 μL of fresh medium, along with 10 μL of Alamar Blue, were added to each well. The cells were incubated for an additional 4 h, after which the absorbance was measured at 570 nm and 630 nm using a microplate reader (Epoch, Biotek). The viability was calculated as stated above. The cell adhesion and morphology for both cell types were inspected via optical microscopy, as presented in Figures S1 and S2.

### 2.8. Drug Release Assay

To evaluate the gentamicin release profile from a PU coating, a protocol was followed over a period of 100 h. Briefly, 10 specimens of EPD gentamicin‐coated specimens (SPU + G) were immersed in 10 mL PBS saline at 37°C to simulate physiological conditions. At predetermined time intervals, 1 mL of the supernatant was collected and replaced with an equal volume of fresh PBS to maintain sink conditions. To quantify the released drug, each 1 mL supernatant specimen was reacted with 0.450 mL of a 2.08 mg/mL solution of ninhydrin, a reagent that binds with gentamicin to form a purple complex. The ninhydrin reaction mixtures were incubated at 85°C for 15 min, and the absorbance was measured at 570 nm according to a previously reported protocol [32]. The cumulative gentamicin release was calculated against a calibration curve prepared from standard solutions. These experiments were conducted in triplicate.

### 2.9. Statistical Analysis

ANOVA tests were implemented to compare specimens without coating (controls) to specimens with EPD coatings. A Tukey test was used with a confidence level of 95% (*p* < 0.05) using the software Origin 2018.

## 3. Results and Discussion

### 3.1. Coating Stability

The performed adhesion test in accordance with ASTM D3359 (Method B) resulted in the complete detachment of the SPU layer (Rating 0B). We contend that this result is a consequence of methodological incompatibility rather than poor interfacial bonding. Said method is primarily designed for rigid, thin coatings (such as paints or inks) on hard substrates. SPU is a low‐modulus elastomer. When the tape is pulled, the SPU undergoes significant elastic deformation and stretching. This concentrates stress at the lattice edges, causing a “peeling” effect that “unzips” the coating, which is a phenomenon well documented for elastomeric films on metal. However, SPU coating stability on titanium can be assessed by other means. For example, conditioning samples in PBS also demonstrate that SPU did not detach from the titanium substrate. In addition, during polarization/electrochemical experiments the SPU layer was stable throughout all experiments. The following section shows the complete characterization of these materials.

### 3.2. Substrate Preparation and EPD

**FIGURE 2:**
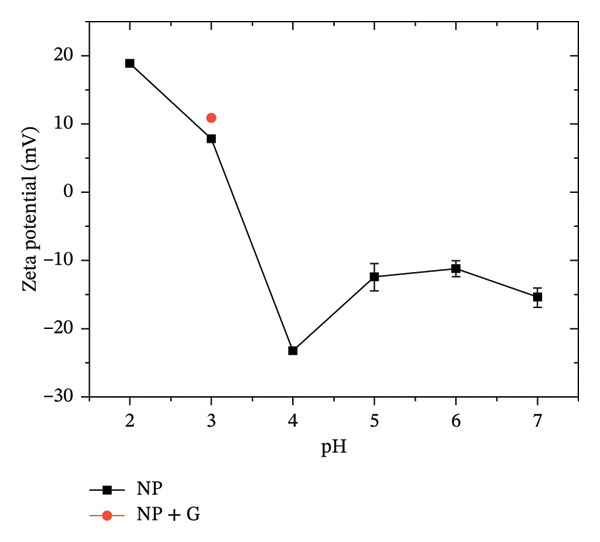
Z potential of NP at different pH, in red the ZP of NP at pH 3 with gentamicin.

The zeta potential of TiO_2_ NP was measured across a range of pH values to assess the suspension’s net surface charge and stability, as shown in Figure 2. Higher absolute zeta potential values correspond to enhanced electrostatic repulsion between particles, which inhibits agglomeration and reduces the hydrodynamic size of the dispersion. The pH at which the zeta potential of particles or surfaces reaches zero is defined as the zero‐point of charge (ZPC), and it is generally advisable to work away from this value to maintain colloidal stability. Below the ZPC, the zeta potential is positive, indicating a predominance of positively charged surface groups; above the ZPC, the zeta potential becomes negative, corresponding to negatively charged NP. In our measurements, the ZPC was reached at pH 3.4. Our results align with those previously reported in [33, 34], where anatase TiO_2_ NP of similar size (25 nm) showed decreasing zeta potential with increasing pH, though with a ZPC of 5.2. This difference in ZPC could be attributed to variations in particle size, surface functional groups, and the specific experimental conditions used in this study. Furthermore, gentamicin exhibits relatively high stability in mildly acidic to neutral conditions (approximately pH 4–7), where it maintains its chemical integrity and antibacterial activity. At lower pH values (pH < 4), gentamicin can still display antibacterial behavior, although partial acid‐catalyzed degradation may reduce its stability. In contrast, alkaline conditions (pH > 7.5–8) promote faster degradation and a pronounced loss of activity [35–37]. When gentamicin is added to the suspension at pH 3, the zeta potential further increases from +7.8 ± 0.4 mV to +10.8 ± 0.4 mV (see Figure 2). The increase in zeta potential (ζ) to +10.8 m V upon the addition of gentamicin suggests adsorption onto the NP surface and a slight improvement of the dispersion stability. Given that gentamicin is also positively charged at this pH due to protonated amine groups, the adsorption mechanism is likely governed by hydrogen bonding, van der Waals interactions, or modifications of the electrical double layer. Consequently, pH 3.0 was selected as the optimal compromise between the physical requirements of the EPD process and the chemical preservation of the gentamicin activity.

In our study, it was found that small amounts of NP and G were deposited on SPU‐coated titanium samples. This behavior is not related to the experimental conditions used (3 min at 25 V) since the parameters chosen here are aligned with the working conditions in other studies, for instance, Santillán et al. [38] used 25 V during 300 s for the EPD deposit of NP on SS316L, whereas Stevanovic et al. [19] deposited mixtures of gentamicin/chitosan/HAP on Ti in acidic conditions during 12 min at 5 V. Finally, Phisbin et al. [18] studied EPD deposition of bioglass/chitosan/gentamicin at 10 V and 400 s. Instead, the limited deposits are attributed to the preloaded SPU layer in the cathode.

In addition, it is known that the use of other polar solvents, such as ethanol or acetone, might improve the deposition yield and prevent water electrolysis [39]; however, these solvents are incompatible with the SPU layer, as they may induce swelling, detachment from the metal, and decrease the gentamicin solubility [40].

The application of a nonconductive layer to metallic electrodes has been explored in previous studies on modified substrates such as rubber, silicon, and ceramics [41–43]. In our study, NP and G deposition on a nonconductive surface (SPU‐coated titanium) is explained as follows. Small permeating currents through the SPU layer indicate that limited electrophoretic motion occurs in the system; however, this effect alone cannot explain the preferential deposition observed at the edges of the porous surfaces. The electrode configuration, with the SPU layer adjacent to the cathode and the liquid electrolyte in between, can be modeled as a capacitor‐like system. Cross‐sectional SEM analysis revealed that the SPU layer has an average thickness of approximately 8.03 ± 1.7 μm (see Figure S3a). At an applied potential of 25 V, the electric field across the dielectric film reaches about *E* = V/d = (3.1 ± 0.7) MV m^−1^, well below the dielectric strength of SPU (typically 20–80 MV m^−1^) [44]. Under these conditions, the SPU acts as a polarized dielectric barrier: a negative charge accumulates at the SPU–cathode interface, inducing a corresponding positive charge at the SPU–electrolyte boundary, confirming that the applied field induces polarization without causing dielectric breakdown. This interfacial polarization creates localized electric potential gradients that attract charged species from the suspension. Consequently, cationic gentamicin molecules and TiO_2_ NPs are driven toward high‐field regions—particularly near pore edges where the local field intensity is amplified—leading to their co‐localization and immobilization on the SPU surface.

### 3.3. Cyclic Polarization

**FIGURE 3:**
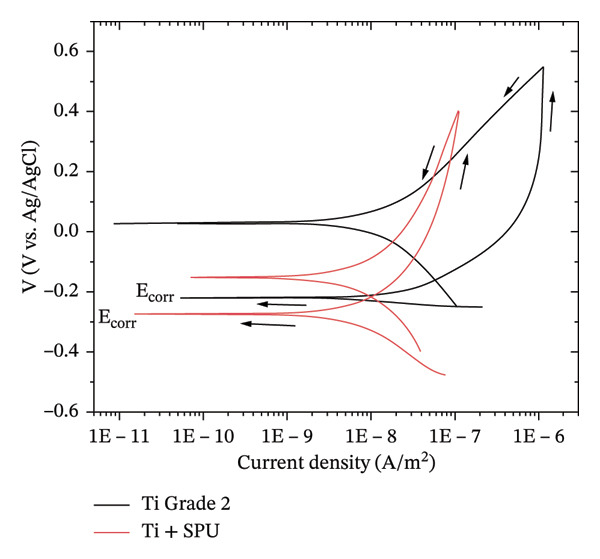
Potentiodynamic polarization curves of bare Ti and SPU‐coated Ti.

Electrochemical experiments were conducted on the bare titanium substrate and coated samples, once the open circuit potential stabilized (see Figure S4a in supporting information). Potentiometric cyclic polarization was performed to evaluate the pitting potential, corrosion rate, and self‐repair capacity of the passive film that can be obtained. Figure 3 shows representative cyclic polarization curves for bare Ti and SPU‐coated Ti. Notably, the polarization curves (Figure S4b and S4c) lacked features typically associated with pitting corrosion [45], suggesting high stability for both surfaces when immersed in the NaCl solution. The electrochemical results reveal distinguishable differences in the corrosion current (i_corr_) derived by fitting the Butler–Volmer equation [46] using the Echem Analyst software from Gamry Instruments. For bare titanium, an average value of 384 ± 253 nA was estimated, confirming the well‐known inherent corrosion resistance of this biocompatible metal. This corrosion resistance is primarily attributed to the spontaneous formation of a stable and adherent titanium oxide layer (mainly TiO_2_) on the surface [47]. Titanium coated with SPU exhibited a low average corrosion current of 11 ± 7 nA. This reduction in i_corr_, compared to the bare substrate, demonstrates the effectiveness of synthetic PU as a protective barrier against corrosive processes. The decrease in i_corr_ values can be attributed to the physicochemical properties of the polymeric coating, which acts as an effective physical barrier limiting the transport of corrosive species to the metal surface.

Using a nonbuffered 3.5% NaCl electrolyte rather than standard physiological buffers (PBS, Hank’s Balanced Salt Solution [HBSS], or Ringer’s solution), it provides a worst‐case scenario for titanium corrosion. However, the use of HBSS or PBS, which contain phosphates and divalent cations (Ca^2+^, Mg^2+^) that provide a secondary layer of protection through the adsorption of phosphate ions and the subsequent precipitation of bone‐like calcium–phosphate minerals [48–50], would be desirable for future studies.

### 3.4. Physicochemical Characterization of NP and Gentamicin Deposits

Figure 4(a) displays the FTIR spectra of the raw materials and the resulting deposits. The spectrum of gentamicin sulfate (G) exhibits characteristic absorption bands consistent with previous reports [51]. Notably, the bands observed at approximately 3421 cm^−1^ and 2963 cm^−1^ are attributed to N–H and C–H stretching vibrations, respectively. The absorption bands at 1619 and 1520 cm^−1^ are related to N–H bending vibrations associated with amide I and amide II groups in the aminoglycoside structure. Additionally, the band at 1128 cm^−1^ indicates S=O vibration, while the band at 619 cm^−1^ is attributed to S–C bending, as previously reported [52]. The SPU spectrum reveals multiple bands in agreement with former studies [53]. Features include an N–H stretching band at 3326 cm^−1^, CH_2_ stretching vibrations between 2936 and 2854 cm^−1^, and a C=O stretching band at 1719 cm^−1^, indicative of urethane groups. A band at 1533 cm^−1^ corresponds to C–N and N–H bending, while a strong band at 1100 cm^−1^ is attributed to asymmetric C–O–C stretching in the polyether backbone. The spectrum of NP shows characteristic features of the anatase phase [54]. A broad band near 3423 cm^−1^ displays O–H stretching, while the band at 1631 cm^−1^ is attributed to Ti–OH bonds from water absorbed by the NP. A band associated with Ti–O–Ti bonds is typically observed between 400 cm^−1^ and 600 cm^−1^. A broad absorption band beginning near the 800 cm^−1^ region encompasses these Ti–O–Ti bonds. Finally, the FTIR spectrum of SPU + NP_G specimens primarily displays features associated with the SPU layer. This suggests that the SPU component is the dominant phase, which effectively masks the individual contributions from the NP and G components.

FIGURE 4(a) Representative infrared spectra; (b) representative Raman spectra of gentamicin powder (G), SPU membranes, TiO_2_ powder (NP), and EPD‐coated specimens with SPU + NP_G.

**Figure:**
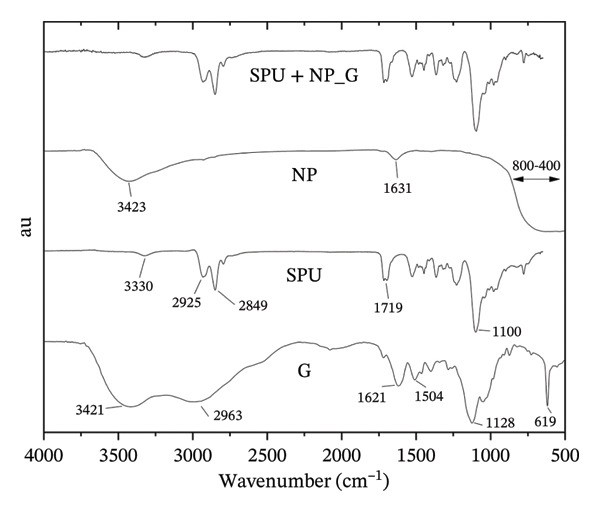
(a)

**Figure:**
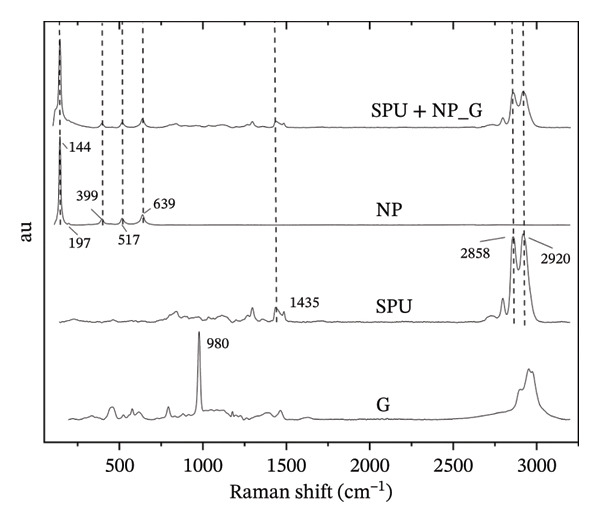
(b)

Raman spectroscopy was employed to identify the chemical components present on the coating surface (Figure 4(b)). The spectrum of neat gentamicin powder displays a prominent mode at 980 cm^−1^, attributed to C–O–C stretching vibrations, consistent with previous reports [55]. The characteristic Raman bands for the TiO_2_ NPs appear at 153, 195, 393, 509, and 635 cm^−1^, in agreement with the vibrational modes reported for the anatase phase [56]. In the SPU spectrum, dominant modes are observed at 2919 and 2860 cm^−1^, corresponding to C–H stretching, alongside bands at 1435 cm^−1^ (aliphatic CH_2_ bending) and 1296 cm^−1^ (C–O and C–N stretching vibrations) [57, 58]. The spectra of the SPU + NP_G coating, which includes NP and the antibiotic, exhibited peaks primarily associated with the SPU matrix and the anatase NP. A band at 980 cm^−1^, associated with G, was not observed, indicating a lower concentration of the substance at the surface. Further evidence of the G deposit was obtained by SEM/EDX.

### 3.5. Structure, Morphology and Quantification of Deposits

AFM was employed to characterize the topography and morphology of the EPD coatings. Phase contrast imaging detects variations in the phase lag between the oscillation of the AFM cantilever and the driving signal as the tip interacts with the specimen surface. These variations highlight differences in the mechanical properties, such as stiffness and adhesion, among the components of the specimen [59, 60]. Therefore, the phase mode enables the differentiation between NP and SPU components. Figure 5 shows representative AFM measurements of 2D and 3D topography and phase contrast of SPU (a, d, g), SPU + NP (b, e, h), and SPU + NP_G (c, f, i) coatings. As can be observed, most SPU layers (Figures 5(a) and 5(d) (> 95%)) form a porous layer derived from the fast evaporation of the solvent (chloroform) during drying. Additionally, it was observed that the pore size and depth vary between specimens. This variability can be attributed to multiple factors, such as slight differences in solvent evaporation rates, ambient humidity, temperature, and the local distribution of SPU during deposition. A distribution of diameters was computed to analyze the pore variability (see S.I. S3b). Several studies in literature have systematically investigated the development of porous structures [61, 62]. Despite the challenges in achieving precise control of porosity, this feature is believed to enhance cell attachment and facilitate improved integration with biological entities.

FIGURE 5AFM measurements of two‐dimensional topography (a–c), three‐dimensional topography (d–f), and phases (g–i) for SPU, SPU + NP, and SPU + NP_G, respectively.

**Figure:**
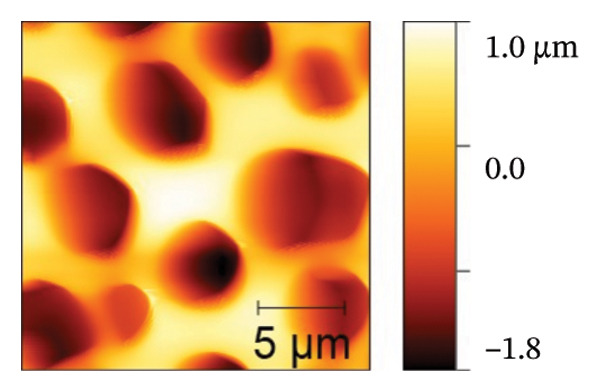
(a)

**Figure:**
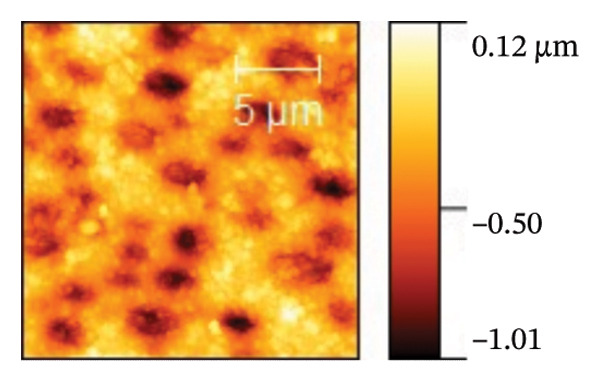
(b)

**Figure:**
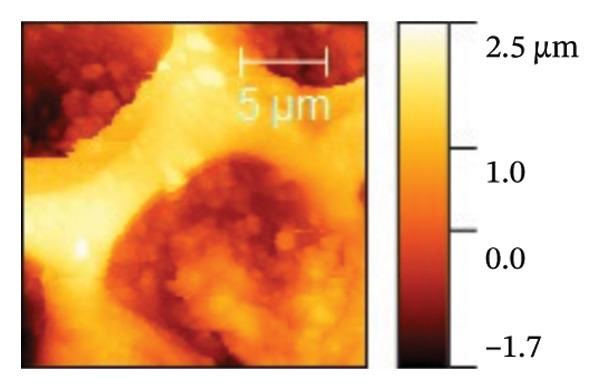
(c)

**Figure:**
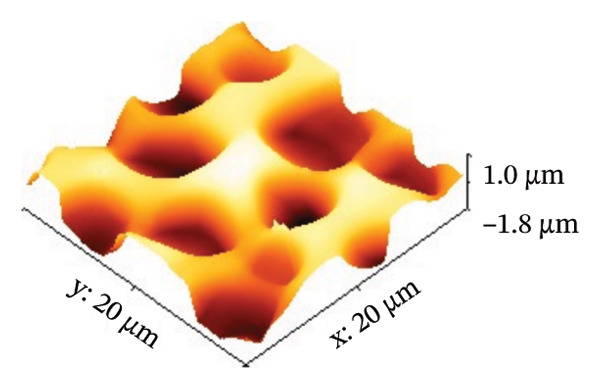
(d)

**Figure:**
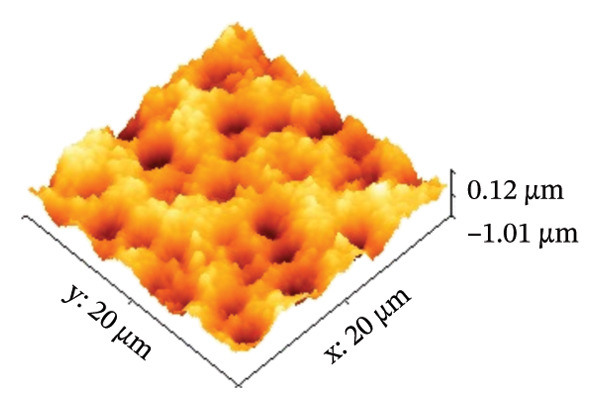
(e)

**Figure:**
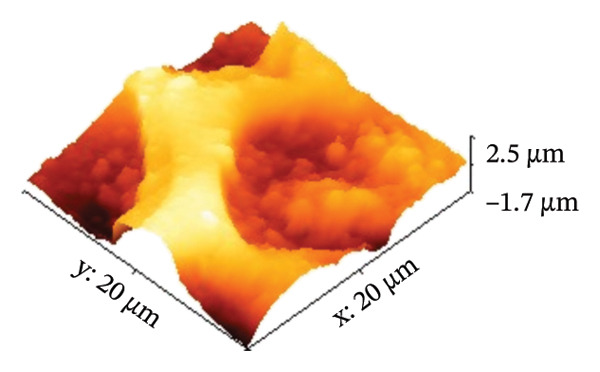
(f)

**Figure:**
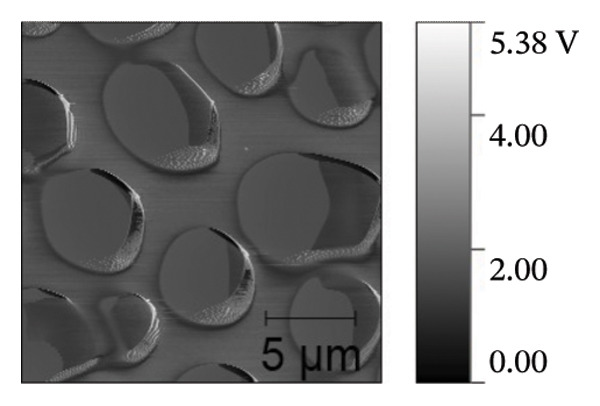
(g)

**Figure:**
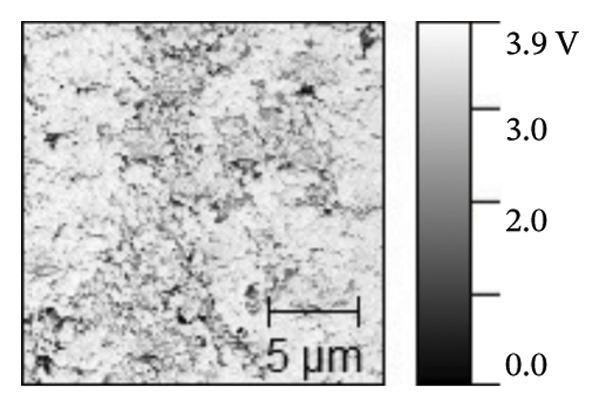
(h)

**Figure:**
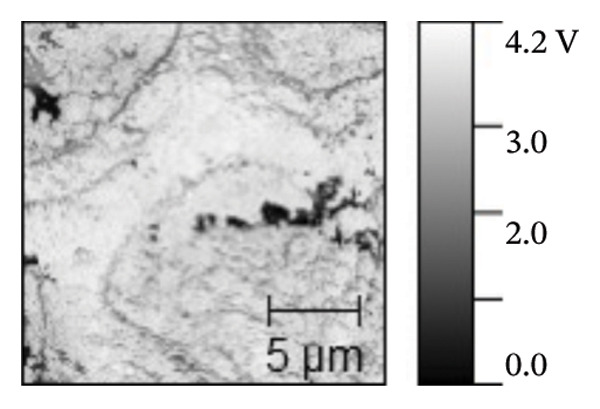
(i)

Specimens coated with SPU from Figure 5(a) and 5(d) indicate pore depths in the order of 1.8 μm with pore diameters of 4.12 ± 0.22 μm. 3D images indicate a smooth surface on the edges of the pores (Figure 5(d)). The phase mode of the SPU (Figure 5(g)) specimen indicated an overall dark shade, indicating the presence of a homogeneous PU layer. Specimens of SPU + NP produced via EPD exhibited a relatively dense granular coating, as shown in Figure 5(b). The particles were observed to cover both the top and bottom of the layer while preserving the overall porosity. A 3D image (Figure 5(e)) depicts a higher roughness on the specimen surface, suggesting the successful deposit of NPs due to electrostatic interaction between the NPs’ positive charge and the still negative cathode charge. The roughness and height parameters are reported in Table 1. It is observed that the Rq index is 2.7 times larger for the SPU + NP (see Figure 5(e)) specimen with respect to the SPU‐only specimen, which agrees with the presence of NP. The phase imaging on SPU + NP reveals white areas corresponding to NP, and the dark areas indicate the presence of SPU (see Figure 5(h)). Using Image J for image analysis, the coverage for different specimens was determined and reported in Table 1. With the addition of gentamicin (Figures 5(c), 5(h), 5(i)) a similar coating of SPU + NP is observed, although a higher roughness was achieved. In this case, both NP and G uniformly adhere to the SPU surface, implying that the deposit of gentamicin does not greatly affect the morphology of the deposit of NP. Studies have shown that nanoscale topography improves cell morphology, cytoskeletal organization, and proliferation in a cell‐specific manner, where an increase from 8.9 nm (bare Ti) to 45.8–51.4 nm (nanotube‐coated specimens) was observed [63]. In our study, an increase in roughness from 29 nm for SPU coated Ti to 78 nm for SPU + NP and finally to 114 nm for SPU + NP_G is expected to have an effect on cell behavior compared to bare titanium. In addition, a higher porosity will render a higher amount of NP and gentamicin due to an increase of effective area. As demonstrated in the next section, cell viability results indicate comparable values between the unmodified and functionalized SPU coatings. This increase in roughness provides a biocompatible interface where the addition of NPs and antibiotics does not compromise metabolic activity. To further explore these interactions, future analyses using SEM for cell morphology or vinculin staining for focal adhesions could be employed to visualize the specific mechanical coupling between the cells and the coating.

**TABLE 1:** AFM parameters for SPU, SPU + NP, and SPU + NP_G.

Specimen	Coverage (%)	Roughness (Rq, nm)	Maximum height (μm)	Maximum depth (μm)
SPU + NP	83 ± 4	78 ± 24	0.1	−1.0
SPU + NP_G	86 ± 15	114 ± 12	2.5	−1.7
SPU	0	29 ± 12	1.0	−1.8

**FIGURE 6:**
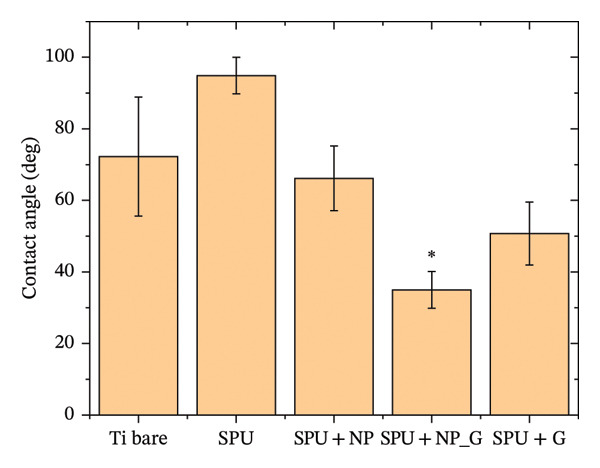
Contact angle of Ti bare, SPU, SPU + NP, SPU + NP_G, and SPU + G.

Surface wettability is a critical factor in evaluating how coatings interact with cells and tissues [64]. CA measurements for the various surfaces are shown in Figure 6. Bare titanium exhibits a CA of 72° ± 16°, while specimens coated with SPU show a CA of 94° ± 5° due to the hydrophobic properties of Tecoflex. The incorporation of NP (SPU + NP) diminishes the CA measured to 66° ± 9°, indicating increased hydrophilicity. This observation aligns with previous studies that report enhanced hydrophilicity following TiO_2_ functionalization [65, 66]. The incorporation of gentamicin (SPU + NP_G) alongside TiO_2_ NPs further enhanced the hydrophilicity of the coating, reducing the water CA significantly to 35° ± 5°. This value is consistent with the low CA observed for the SPU + G specimen and falls within the optimal range (35°–80°) reported for surfaces intended for biocompatibility studies [67]. Based on this criterion, the SPU + NP_G specimen offers optimal conditions for promoting biological interaction.

To relate the CA measurements with the roughness data obtained from AFM, the Wenzel model is often used. This principle states that increasing the roughness of a hydrophobic surface typically amplifies its water repellency [68]. In this case, while the base PU is slightly hydrophobic, the addition of NPs and antibiotics caused a significant transition to a hydrophilic state, despite the concurrent increase in surface roughness. The observed reversal of this trend provides strong evidence that the chemical functionality of the antibiotic (rich in polar groups) dominates the surface energy, successfully “switching” the interface from a hydrophobic state to a bio‐receptive, hydrophilic one. Furthermore, the inherent porosity of the SPU layer must be considered, as it likely transitions the surface behavior from simple Wenzel wetting to capillary‐driven imbibition. This porosity not only contributes to the significant drop in CA by increasing the effective internal surface area but also serves as a high‐capacity reservoir for the antibiotic.

SEM imaging was used to observe the morphology features of the EPD coatings. Figures 7(a) and 7(d) show the SEM micrographs of NP powder, as indicated by the supplier, the particle size of the NP is 21 nm, which is consistent with the obtained images. Figure 7(b) and 7(e) portrays the presence of porous surfaces densely coated with NPs (SPU + NP).

FIGURE 7SEM micrographs of (a and d) powder of NP at two different magnifications, (b and e) Ti coupons with SPU + NP coating at two different magnifications, and (c and f) Ti with SPU + NP_G coating at two different magnifications.

**Figure:**
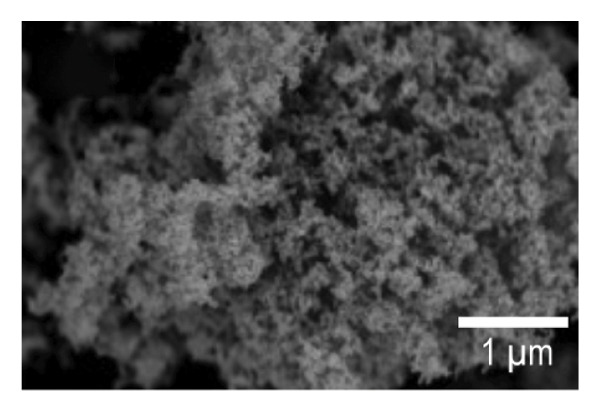
(a)

**Figure:**
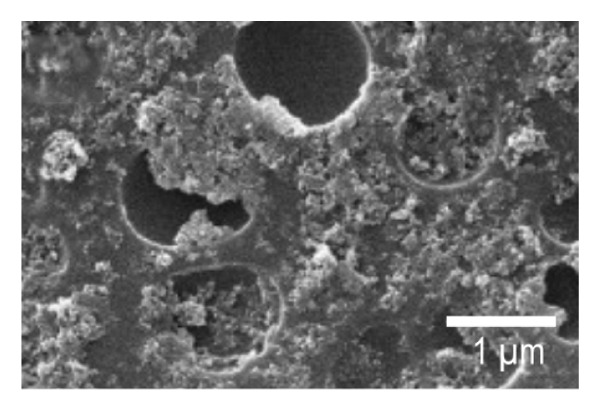
(b)

**Figure:**
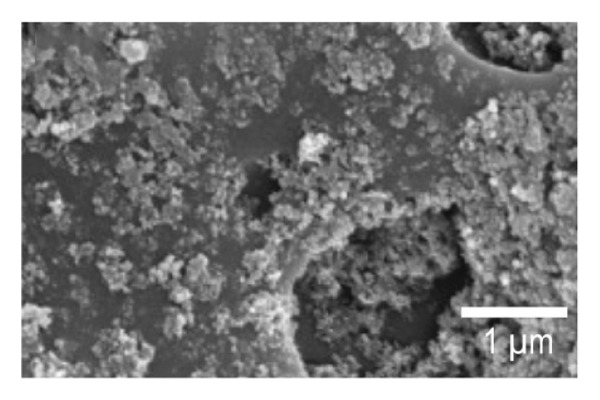
(c)

**Figure:**
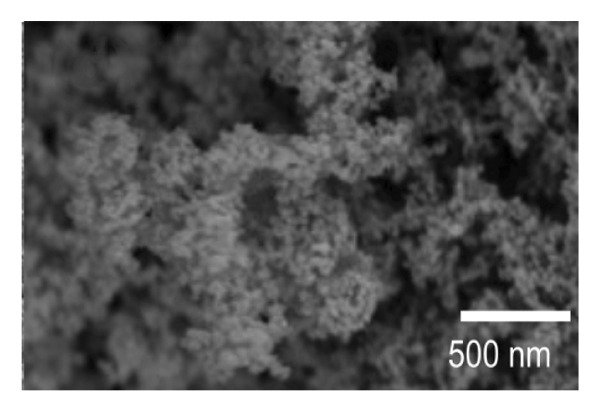
(d)

**Figure:**
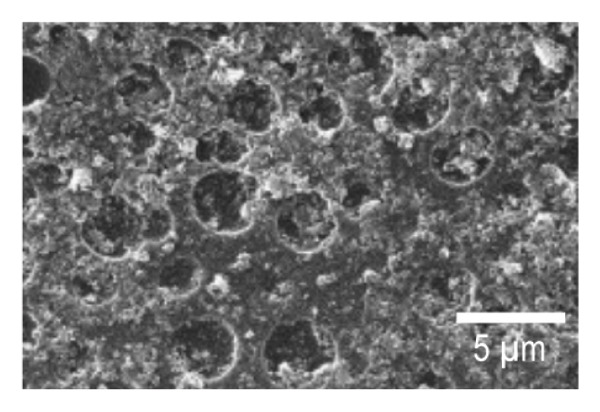
(e)

**Figure:**
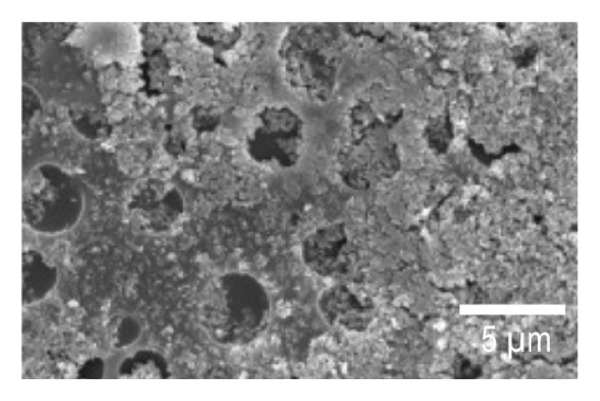
(f)

Similarly, EPD coatings with antibiotics (SPU + NP_G) shown in Figures 7(c) and 7(f) exhibit a PU layer coated with NPs and with large agglomerates present. SEM results agree with AFM data, where larger surface roughness is caused by the higher agglomeration degree of the antibiotic‐containing specimen. Cross‐sectional SEM imaging (Figure S3a) was used to evaluate the SPU base layer, revealing a nonmonolithic matrix with an average thickness of 8.03 ± 1.7 μm with no apparent microcracks.

**FIGURE 8:**
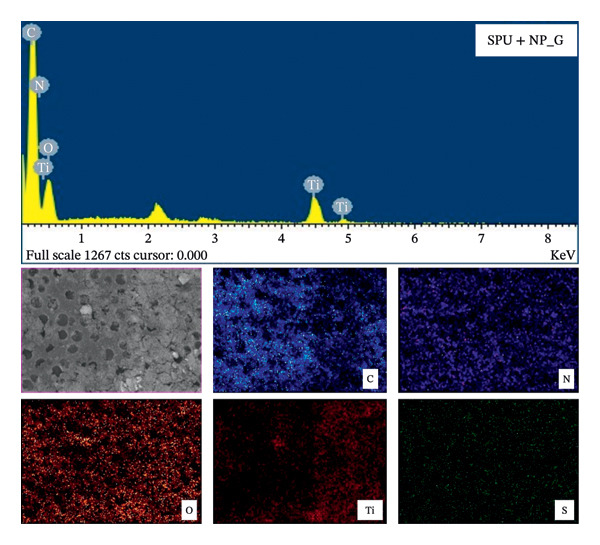
EDX qualitative mapping of EPD deposits. Each frame depicts the localization of an element in the coating.

SEM imaging alone cannot provide definitive evidence of the presence of antibiotic G in the EPD coatings. To address this, EDX mapping was conducted to visualize the elemental distribution (see Figure 8). The detection of sulfur (S) in the EDX mapping indicates the presence of gentamicin sulfate, as the sulfur originates from the sulfate ions present in the salt form commonly used to enhance the antibiotic’s stability. For the specimens studied, it was observed that S is distributed evenly to the surface and is not necessarily correlated with the location of NP. This suggests that the gentamicin is not exclusively adsorbed to the NP surfaces but is also incorporated throughout the SPU matrix. Quantitative EDX analysis (see Table 2) on SPU + NP_G showed that the most abundant elements were carbon (C), nitrogen (N), and oxygen (O) due to the SPU and NP composition. Additionally, a Ti content of 5.22 weight % was measured, confirming the successful incorporation of the NPs within the deposit.

**TABLE 2:** Quantitative EDX of prevalent elements on EPD specimen SPU + NP_G, pristine SPU, gentamicin powder, and TiO_2_ powder.

Element	SPU + NP_G wt %	SPU + NP_G at %	SPU wt%	SPU at %	G wt %	G At%	NP wt %	NP at %
C K	42.56	49.55	71.61	76.56	43.85	53.62	ND	ND
N K	26.45	26.41	4.75	4.37	ND	ND	ND	ND
O K	25.77	22.52	23.65	19.07	44.93	41.24	57.85	80.43
S K	ND	ND	ND	ND	11.23	5.14	ND	ND
Ti K	5.22	1.52	ND	ND	ND	ND	42.15	19.57
Total	100	100	100	100	100	100	100	100

SEM/EDX results were used also to explain NP deposition. SEM did not show the presence of a crack in the SPU‐coated titanium metallic coupons, while EDX analysis did not show Ti on the surface of the coating, and only C, O, and N were detected. This suggests that even when porosity is present, there is a dense layer on the bottom that fully covers the metal. Therefore, an electric field penetrates this insulating coating to allow TiO_2_ NP deposition.

For SPU + NP_G specimens, no traces of the S element were quantified, which may be attributed to the limitations of EDX detection efficiency for lighter elements, since the sulfur atom in gentamicin (*Z* = 16) produces a much weaker signal compared to titanium (*Z* = 22). Furthermore, EDX is a surface‐sensitive technique where signals are highly element‐dependent. TiO_2_ NPs are discrete, inorganic solids that tend to remain at or near the surface, providing a strong and easily detectable titanium signal. Gentamicin, by contrast, is a small organic molecule (c.a. 3% sulfur) likely distributed within or partially absorbed into the SPU matrix, resulting in reduced surface exposure. However, as demonstrated by the mapping, gentamicin is confirmed to be present in the coatings.

A similar elemental quantification was reported for iron‐based coatings incorporating gentamicin and polyethylene glycol, where the atomic percentage (At. %) of sulfur was 0.4% [69], indicating significantly lower abundance compared to other elements. In that study, the coating was applied via deep coating using an initial gentamicin concentration of 6 mg/mL. In contrast, our study utilized a lower initial concentration of gentamicin (1 mg/mL); therefore, a proportionally reduced sulfur content is anticipated.

**FIGURE 9:**
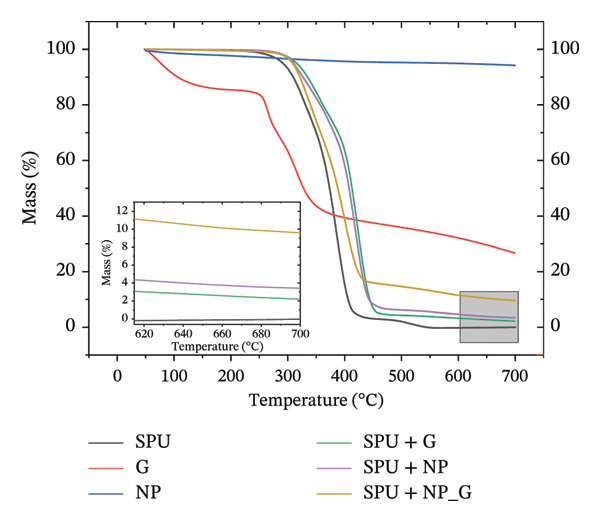
TGA thermograms of EPD coatings; the inset shows a zoom in of the area highlighted in gray. SPU and NP are specimens that correspond to pristine materials. The left axis is a guide to the eye of the mass axis.

TGA was performed to determine the mass of NP and G coated at the SPU surface, and the results are shown in Figure 9. This technique is highly suitable for detecting small changes in mass, typically in the microgram range. Results are summarized in Table 3. The thermograms of SPU specimens showed a complete weight loss of SPU of 100% at 550°C, similar to other reported findings [70]. The thermogram of the SPU specimen reveals three distinct decomposition events. The first decomposition occurs between 225°C and 330°C, accounting for a 20% mass loss, which can be attributed to the degradation of the rigid segments. The second decomposition takes place between 350°C and 420°C, corresponding to a 77% mass loss, primarily associated with the degradation of the flexible segments in the PU structure. The final decomposition is observed between 470°C and 570°C, representing a 3% mass loss, likely due to the breakdown of residual char or the degradation of carbonaceous material formed during earlier stages. This final stage signifies the complete thermal decomposition of the PU. NP does not show a degradation window, with a total weight loss of 2% attributed to water molecules adsorbed on the surface, where similar results have been found in literature [71, 72]. Degradation of gentamicin showed significant weight loss below 150°C, between 250°C and 370°C, resulting in a slight decrease from 370°C onwards for a final weight loss of 73%, where the obtained thermogram agrees with other reported results [73]. Based on the thermogravimetric behavior of the individual species, the experimental mass loss of the composite specimen (88.8%) and the residual mass (9.6%) were used to estimate the relative contributions of each component. By solving a normalized system of equations incorporating the specific decomposition profiles of each species (see S5 on supporting information), the estimated mass fractions were approximately 61.7% for SPU (completely decomposed), 8.5% for G (6% mass loss), and 29.8% for TiO_2_ NP (73% mass loss). The surface densities of the components were estimated by dividing the individual average masses by the coating area (1.54 cm^2^). From the calculations, a surface density of 0.21 ± 0.08 mg/cm^2^ for NP and 0.78 ± 0.14 mg/cm^2^ for gentamicin was estimated when deposited with EPD on the SPU layer individually. When deposited simultaneously (SPU + NP_G), a competing effect occurs between each of the coating components, resulting in lower surface densities (with respect to the single deposits) corresponding to 0.19 ± 0.03 mg/cm^2^ for gentamicin and 0.11 ± 0.01 mg/cm^2^ for NP. The amount of NP and gentamicin deposited is different despite both carrying positive charges. For NPs, the zeta potential is the primary driving force for electrophoretic mobility. In contrast, the deposition of gentamicin is governed by its degree of ionization, determined by its pKa values (pKa = 8.2–9.8), which results in a higher molar deposition rate. However, it must bear in mind that the real surface area is higher due to SPU porosity, and this will underestimate the corresponding surface density.

**TABLE 3:** Quantitative TGA analysis of deposited mass.

Specimen	Total initial mass (mg)	Final mass (mg)	Total mass loss (%)	Initial mass SPU (mg)	Initial mass NP (mg)	Initial mass G (mg)	TiO_2_ (mg/cm^2^)	G (mg/cm^2^)
SPU	6.20 ± 0.40	0 ± 0	100	6.20 ± 0.40	—	—	—	—
G	5.40 ± 1.20	1.46 ± 0.25	73	—	—	5.40 ± 1.20	—	3.51 ± 0.78
NP	3.07 ± 0.80	0.18 ± 0.02	6	—	3.07 ± 0.80	—	1.99 ± 0.52	—
SPU + NP	5.02 ± 1.90	0.22 ± 0.05	97	4.69 ± 1.78	0.33 ± 0.12	—	0.21 ± 0.08	—
SPU + NP_G	2.54 ± 0.30	0.24 ± 0.04	93	2.07 ± 0.24	0.17 ± 0.02	0.30 ± 0.04	0.11 ± 0.01	0.19 ± 0.03
SPU + G	7.45 ± 1.42	0.21 ± 0.05	98	6.67 ± 1.17	—	0.78 ± 0.14	—	0.51 ± 0.10

### 3.6. Antimicrobial Susceptibility Assay

To assess the antibacterial activity of the EPD coatings, a disk diffusion assay was performed against the Gram‐positive *S*. *aureus* and the Gram‐negative *E*. *coli* (see Figure 1(a) and Figure S6 in S.I.).

Ti disks coated with SPU do not show bacterial inhibition for any of the strains, while Tecoflex is not biologically inert but does not generate any effect on bacteria. EPD‐coated disks with NP (SPU + NP) did not generate any antibacterial response in any of the strains. As a reference, a specimen with a known amount of gentamicin (0.1 mg) was incubated on a Ti disk coated with SPU (SPU + G_ref_); these specimens showed inhibition halos of 35.7 ± 2.6 mm for *E*. *coli* and 29.5 ± 2.4 mm for *S*. *aureus*. It is known that gentamicin is a broad‐spectrum antibiotic against Gram‐positive and Gram‐negative bacteria; these results are in agreement with other reported assays with gentamicin on these strains [74, 75].

Specimens with gentamicin‐only EPD deposits (SPU + G) also generated an inhibitory response, although the area zones were smaller, 27 ± 5.7 mm for *E. coli* and 23.3 ± 4.9 mm for *S. aureus*. From this result, it is observed that gentamicin preserved its antibacterial effect after electrophoretic treatment. The smaller areas are attributed to a lower amount of gentamicin deposited during EPD, as the previous results showed. Specimens produced by EPD of both NP and G (SPU + NP_G) also showed an inhibitory response to the two bacterial strains comparable to the case of having only gentamicin deposited, with 22.0 ± 2.0 mm for *E. coli* and 28.3 ± 3.7 mm for *S. aureus*. These results confirm that the presence of TiO_2_ NPs does not compromise the antibacterial properties of the coatings. Similar results were obtained when gentamicin was incorporated by EPD, with primary use for orthopedics [18, 76, 77].

Based on the inhibition of both strains in the agar diffusion assays, *S. aureus* was selected as the representative model for subsequent microdilution analysis due to its high clinical relevance in surgical site infections; see Figure 1(b). A control specimen of MHB was used for comparison. Interestingly, specimens that tested bare Ti showed no increase in bacterial population against the control, while the disk coated with PU showed comparable bacterial growth with respect to the control. Similarly, the specimen coatings with NP produced by EPD (SPU + NP) did not show an antibacterial response as in the previous agar assay. The extract from reference specimens containing a known amount of gentamicin (100 μg) demonstrated a significant reduction in bacterial population. Similarly, specimens produced by EPD with gentamicin (SPU + G) and those with both NPs and gentamicin (SPU + NP_G) also exhibited lower OD, indicating a significant decrease in bacterial populations. Thus, the results from the agar disk assay are consistent with those obtained from the microdilution assay. The lack of statistical difference in the halo test is attributed to the diffusion‐limited nature of the agar matrix in addition to diffusing molecule size and the inability to distinguish between a bacteriostatic and a bactericidal effect. In contrast, the liquid‐phase turbidity test provides higher sensitivity to the combined effect of the TiO_2_ NP and G. Collectively, these complementary methods validate that gentamicin remains with antibacterial activity.

The antibacterial assays showed an acceptable activity of the coatings against *S. aureus* and *E. coli*. It was demonstrated that gentamicin retained its antibacterial activity after EPD, while in previous studies we showed that other antibiotics like nisin did not behave as antibiotics after EPD, highlighting the need for further optimization [29].

The antibacterial performance of the coating is driven by gentamicin’s ability to target both the structural and metabolic components of the bacteria. The drug displaces essential divalent cations from the cell membrane, triggering localized permeabilization. Once internalized, gentamicin irreversibly targets a specific ribosomal subunit, causing lethal errors in protein synthesis that effectively arrest bacterial proliferation [78].

### 3.7. Cell Viability Assays

Cell viability studies of fibroblasts and DPMSCs are presented in Figures 10(a) and 10(b), respectively. Fibroblast cells play a critical role in mediating osseointegration and the remodeling of soft tissues surrounding implants [79]. Therefore, it is essential to study the interaction of the obtained coating with such cells. In these studies, the cell viability of fibroblasts is not affected by the particles and antibiotics loaded by EPD, as at 48 h, the viability of the specimen SPU + NP_G is slightly lower than the SPU‐coated specimen, and at 72 h, the cell viability recovered. Gentamicin has been shown to affect fibroblasts in various ways depending on the administered dose. At low concentrations (1–10 μg/mL), it can stimulate cell growth and increase longevity in human lung fibroblasts, while higher concentrations above 100 μg/mL were toxic, but cell growth was still observed [80]. A possible mechanism for cytotoxicity specific to fibroblasts was investigated in [81], where the authors found that gentamicin forms complexes with melanin and thereby inhibits collagen biosynthesis. Also, other researchers have shown that bioactive TiO_2_ coatings facilitate soft tissue integration and fibroblast attachment [82, 83].

FIGURE 10Cell viability of (a) fibroblasts and (b) DPMSCs under exposure to EPD coating extracts at 24, 48, 72 h, and 7 days. Data are presented as mean ± SD (*n* = 3). Statistical analysis was performed using one‐way ANOVA followed by Tukey’s post hoc test. *p* < 0.05 was considered significant.

**Figure:**
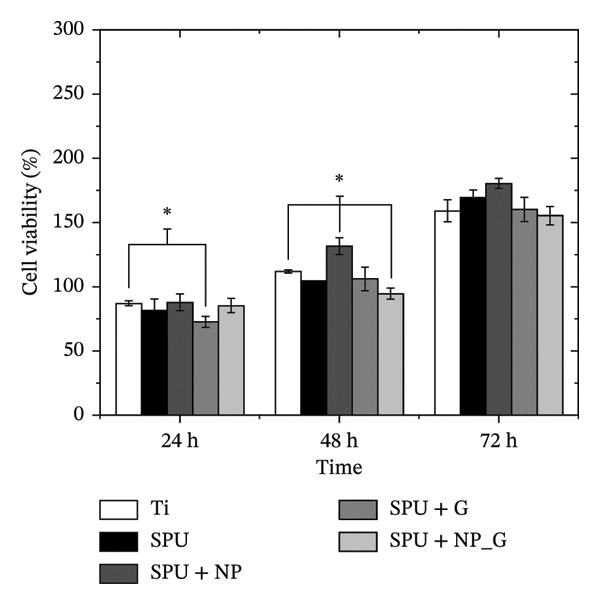
(a)

**Figure:**
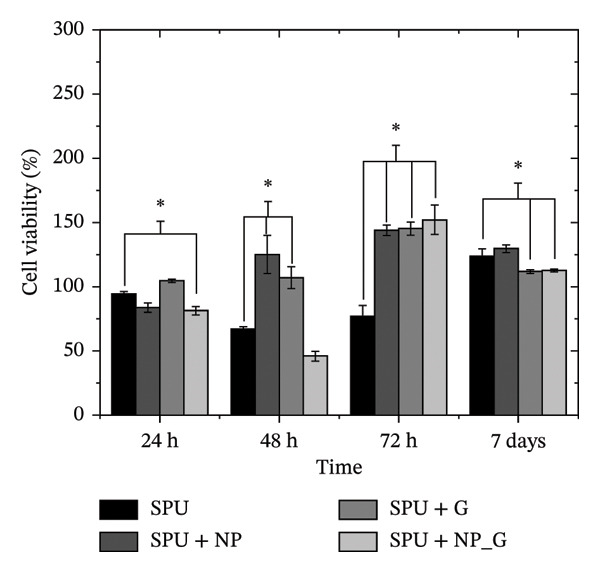
(b)

A subset of specimens was evaluated for DPMSC viability (which can be differentiated into osteoblast lineage) over an extended period of 7 days. After 24 h, the viability of all EPD‐produced specimens was comparable to that of Ti discs coated only with SPU, except for the SPU + NP_G specimens, which exhibited reduced viability. At 48 h, specimens with G only and NP only increase viability, while specimens with both compounds, NP and G, have a reduced viability. However, at 72 h, the viability of SPU + NP_G specimens increased beyond that of the control, reversing the initial trend. By Day 7, all specimens had stabilized, with viabilities exceeding 100%. In this regard, it has been reported that gentamicin has negative effects on mesenchymal stem cells, for instance, from equine [84] and human bone marrow [85] at different concentrations of the antibiotic, 150 mg/mL and 50–200 μg/mL, respectively. Interestingly, in human bone marrow MSCs, gentamicin did not have an adverse effect on viability but did inhibit cell proliferation at concentrations larger than 100 μg/mL. Furthermore, dental pulp stem cells showed increased proliferation in the presence of antibiotics [86]. As estimated from the TGA data, the mass deposited was in the order of 200–300 μg of G in combination with NP. However, EPD specimens did not affect the viability of DPMSCs or the viability of fibroblasts. This suggests a positive effect by the simultaneous deposition of particles and antibiotics. It would be interesting, however, to investigate the susceptibility of alkaline phosphatase, which is a marker for osteogenic differentiation, since it has been reported that gentamicin might reduce the expression of this marker in specific cell lines [87].

One concern regarding the formation of the SPU film is the use of chloroform as the solvent. However, cytotoxicity assays showed no detectable adverse effects attributable to residual chloroform. This is likely due to the prolonged evaporation time employed during specimen preparation and the very thin nature of the polymer films, which facilitate complete solvent removal prior to biological testing.

### 3.8. Drug Release Profile

**FIGURE 11:**
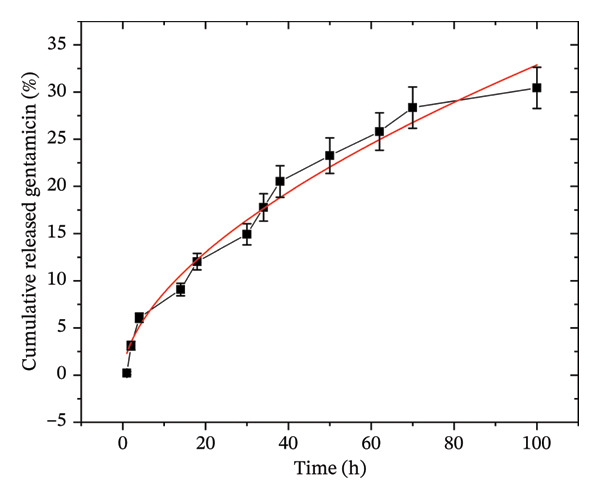
Time release profile of gentamicin from SPU + G surfaces in PBS at 37°C. Results are presented as the percentage of cumulative release *M*_*t*_/*M*_*∞*_; the red continuous line shows the fitting according to the Korsmeyer–Peppas model.

The initial release profile of gentamicin from the EPD coatings was evaluated for the short term using an indirect quantification method based on UV–Vis spectroscopy. Specifically, a ninhydrin assay was employed to determine the gentamicin concentration in solution (see Figure S7a in the supporting information). As detailed in the Methods section, coated titanium (Ti) disks were immersed in PBS, and the release of gentamicin was monitored in triplicate at specific time intervals (see Figure 11). Experiments were performed in triplicate. This approach facilitated the assessment of gentamicin release kinetics over time, providing insights into the drug’s sustained release. In controlled drug release studies, the Korsmeyer–Peppas model [88, 89] is commonly used to describe the mechanism of drug release from polymeric matrices. This semi‐empirical model relates the cumulative drug release M_t_/M_∞_ over time to a power‐law expression:

(2) MtM∞=ktn,

where *M*_*t*_ is the cumulative amount of drug release at time *t*, *M*_*∞*_ is the total amount of drug released at equilibrium, *k* is the kinetic constant incorporating structural and geometric characteristics of the drug delivery system, and *n* is the release exponent, which indicates the release mechanism. In the case of thin films, depending on the *n* value of the fitting, the release mechanism could be quasi‐Fickian (*n* < 0.5), Fickian (*n* ≈ 0.5), anomalous (0.5 < *n* < 1), or polymer relaxation/degradation (*n* > 1).

Our data demonstrated a high correlation with the Korsmeyer–Peppas model (*R* ^2^ = 0.97) with a release exponent of *n* = 0.56 ± 0.01, which indicates quasi‐Fickian transport. Some deviations from ideal Fickian behavior may arise due to surface interactions, polymer relaxation, or swelling, though these effects are likely minimal. Given the hydrophobic and nonswelling nature of the polymer and the presence of non‐interconnected porosity, the deviation from ideal Fickian behavior (*n* = 0.5) can be attributed to physical entrapment by the soft segments of the SPU (for Tecoflex, PTMEG above its glass transition temperature) or drug confinement within isolated pores and transient electrostatic adsorption onto charged pore walls. Given that the films were prepared by EPD, residual surface charges or oriented dipoles introduced during deposition may contribute to weak electrostatic interactions with the drug molecules. These localized electrostatic potentials introduce additional resistance to diffusion/release by creating charge‐dependent retention sites, resulting in a time‐dependent effective diffusivity governed by desorption rather than polymer relaxation. Similar effects of surface charge heterogeneity on molecular mobility and interfacial interactions have been discussed in the context of biofunctionalized porous materials by Zhang et al. [90], where electrostatic modulation at the pore surface was shown to influence transport and binding dynamics in complex scaffolds. Additionally, we observed that 15% of gentamicin is released within the first 40 h, and by 100 h, 30% of the drug has been released. Similar findings have been reported for the release of diclofenac from PU films [91], where diffusion was identified as a key mechanism controlling drug release, together with mechanisms dependent on drug concentration.

The release kinetics of SPU + G specimens were analyzed as previously described. However, specimens containing both NP and antibiotics (SPU + NP_G) could not be directly assessed due to overlapping absorbance signals. At certain wavelengths, the measured absorbance results from a combination of the ninhydrin signal and the background absorbance of TiO_2_, as characterized in Figure S7b on S.I. To further investigate this, an additional SEM analysis was performed on SPU + NP_G specimens before and after incubation at 37°C for 24 h in PBS (Figures S8a and S8b). While minor liberation of a few loosely bound surface particles occurs, the majority of NP remains immobilized on the SPU substrate. The observations revealed not only a stable coating but also that the first particles to dissociate from the SPU surface were those located within the pores, while the particles along the edges remained more compact and adhered to the polymer structure. For samples that contain NP and G, it is expected that NP will affect gentamicin release in the following manner: NPs act as physical spacers that increase the effective tortuosity and path length for drug diffusion without forming covalent bonds. This ensures that the gentamicin remains chemically stable and mobile within the surface‐accessible voids.

Based on the gentamicin absorbance calibration curve (Figure S7a), the gentamicin concentration in the extraction medium at equilibrium (100 h) was (170 ± 60) μg/mL, corresponding to an approximate range of 110–230 μg/mL. As mentioned in the previous section, in vitro studies indicate that fibroblast cell lines exposed to gentamicin concentrations above > 100 μg/mL toxic effects may occur, although residual cell growth is still observed [80]. Other mesenchymal stem cells from human bone marrow at 50–200 μg/mL show limited cytotoxic responses [86]. Although the total gentamicin released suggested some cytotoxic effect on fibroblasts and DPMSCs, our cytotoxicity measurements after 72 h demonstrated a noncytotoxic response, with cell viability values exceeding 100% for both cell lines, indicative of proliferative behavior under the tested conditions. This observation indicates that the released gentamicin did not adversely affect cell viability over extended exposure times, as this does not correspond to dynamic physiological conditions.

The present results are restricted to a short‐term release evaluation (100 h), but extended‐release studies are required to characterize a complete gentamicin detachment from the coating. Furthermore, testing in physiologically relevant media (simulated body fluid) and subsequent in vivo evaluation will be essential to determine the applicability of the assay and to assess potential interactions or interferences, particularly in systems containing TiO_2_ NPs. Additionally, to fully validate long‐term safety, a dedicated study on the combined liberation of NP and gentamicin under physiological conditions is required.

## 4. Conclusions

In this work, the EPD of gentamicin and TiO_2_ anatase NPs was performed on PU‐coated Ti disks. This approach differs from conventional EPD due to the presence of a dielectric SPU interface. While traditional EPD involves particle discharge on a bare conductive surface, our configuration utilizes a pre‐applied, nonmonolithic SPU film (≈8 μm). The data suggest that the electric field facilitates the transport of TiO_2_ and gentamicin into the film’s pores while simultaneously ensuring consistent coverage across edges and complex geometries. This confirms that the SPU layer allows for particle sequestration within the matrix, providing a method to functionalize pre‐existing polymer coatings to enhance corrosion resistance, antibacterial activity, and sustained drug release.

Our study shows that combining anatase NPs with gentamicin rendered a noncytotoxic effect while retaining gentamicin‐related antimicrobial activity. In this work, TiO_2_ NPs improved fibroblast and MDPSC viability compared to SPU alone at 72 h. In parallel, specimens coated with NP and antibiotics exhibit comparable cell viability. SEM and AFM analyses revealed up to 86% of surface coverage in SPU + NP_G specimens, accompanied by an increase in roughness (≈Rq = 114 nm). TGA enabled the quantitative estimation of the relative mass contribution of each component by comparing the total and residual masses after controlled thermal degradation. While SPU polymer exhibited complete thermal degradation, gentamicin and TiO_2_ showed partial stability, with residual masses of 27% and 94%, respectively. These findings have important implications for drug delivery and surface functionalization: while SPU serves as a protective matrix, TiO_2_ remains as a stable nanostructured component, and gentamicin provides an antimicrobial action through a sustained release mechanism.

This study demonstrates significant potential for functionalizing metallic surfaces used as implants. In systems such as titanium pins or screws, the combination of antibiotic elution and cell adhesion is critical to prevent infections and promote tissue remodeling. Furthermore, this technique is compatible with a wide range of polymeric coatings on metallic surfaces and biomolecules in aqueous media.

## Author Contributions

Fabiola A. Gutiérrez‐Mejía worked on the conceptualization, experimenting, formal analysis, investigation, methodology, resources, supervision, validation, and writing–original draft preparation. Claudia Vásquez‐López, Luis Díaz‐Ballote, Claribel Huchin‐Chan, Arely M. González‐González, and Rossana F. Vargas‐Coronado worked on methodology, experimenting, formal analysis and review and editing. Fabiola E. Villa‐de‐la‐Torre, Victor E. Arana‐Argaez, Raymundo Cruz‐Pérez, and Raúl Rosales‐Ibáñez worked on conceptualization, supervision and review and editing. Juan V. Cauich‐Rodríguez worked on funding acquisition, project administration, conceptualization, supervision, and writing–review and editing.

## Funding

We thank the Secretaría de Ciencia, Humanidades, Tecnología e Innovación (SECIHTI, former CONAHCYT) for funding this project through the grant numbers 332738 (Fabiola Azucena Gutiérrez Mejía) and 637410 (Claudia Vásquez López). Additionally, this research was funded by CONACYT grant numbers 1360 (Fronteras de la Ciencia) and 248378 (Atención a Problemas Nacionales).

## Conflicts of Interest

The authors declare no conflicts of interest.

## Supporting Information

Figure 1. Morphological progression of DPMSCs; Figure S2. Fibroblasts cytocompatibility of SPU + NP_G coatings over 3 days; Figure S3. Cross section and pore size of a sample of Ti coated with SPU; Figure S4. Measurements of EOCP and CP of Ti and Ti + SPU samples; Table S5. Calculation of mass deposited from TGA measurements; Figure S6. Agar plates for antibacterial activity; Figure S7. a,b calibration curve of gentamicin by the ninhydrin colorimetric assay, complexes with ninhydrin produce purple color depending on the gentamicin concentration. c. Calibration curve of TiO2 nanoparticles; Figure. S8 a. A sample coated with EPD dried, b. A sample after 24 h of incubation in phosphate‐buffered saline (PBS).

## Supporting information


**Supporting Information** Additional supporting information can be found online in the Supporting Information section.

## Data Availability

The data that support the findings of this study are available in the supporting information of this article.
