# Supplementary material for: Anatase–Gentamicin Electrophoretic Coatings on Polyurethane‐Modified Titanium: Antibacterial Performance, Biocompatibility, and Drug Delivery
Source: Int J Biomater. 2026 Apr 28;2026:7643259. doi: 10.1155/ijbm/7643259 (PMC13122730; doi:10.1155/ijbm/7643259)
Supplement: Supplementary file 1 — Supporting Information Additional supporting information can be found online in the Supporting Information section. [file IJBM-2026-7643259-s001.docx]

Supplementary information

**Anatase–Gentamicin Coatings on Polyurethane-Modified Titanium: Antibacterial Performance, Biocompatibility, and Drug Delivery**

*Fabiola A. Gutiérrez-Mejía^1^, Rossana F. Vargas-Coronado^1^, Claudia Vásquez-López^2^, Luis Díaz-Ballote^3^, Claribel Huchin-Chan^4^, Fabiola E. Villa-de-la-Torre^4^, Víctor E. Arana-Argaez^4^, Raúl Rosales-Ibañez^5^, Arely M. González-González^6^, Raymundo Cruz-Pérez^7^, Juan V. Cauich-Rodríguez^1^,**

^1^ Materials Department, Centro de Investigación Científica de Yucatán (CICY), Mérida, Yucatán, Mexico

^2^  Physics Department, Universidad de Sonora (UniSon), Hermosillo, Sonora, México

^3^ Applied Physics Department, Centro de Investigación y Estudios Avanzados del IPN Unidad Mérida (CINVESTAV), Mérida, Yucatán, México.

^4^ Chemistry Faculty, Universidad Autónoma de Yucatán (UADY), Mérida, Yucatán, México.

^5^ Laboratory of Tissue Engineering and Translational Medicine, Faculty of Higher Education Iztacala, National Autonomous University of Mexico (UNAM), México City, México

^6^ Research Laboratory in Nano and Dental Biomaterials, Faculty of Higher Studies Iztacala, National Autonomous University of Mexico (UNAM), México City, México

^7^ Department of Infectomics and Molecular Pathogenesis, Center for Research and Advanced Studies (CINVESTAV), México City, México.

*Correspondence: J.V.C.R jvcr@cicy.mx; Tel.: (+52) 999 942 83 30 ext. 424

Contents

Figure S1. Morphological progression of DPMSCs

Figure S2. Fibroblasts cytocompatibility of SPU+NP_G coatings over 3 days

Figure S3. Cross-section and Pore size of Ti coated with SPU

Figure S4. Measurements of EOCP and CP of Ti and Ti+SPU samples

Table S5. Calculation of mass deposited from TGA measurements

Figure S6. Agar plates for Antibacterial activity.

Figure S7. a,b calibration curve of gentamicin by the ninhydrin colorimetric assay, complexes with ninhydrin produce purple color depending on the gentamicin concentration. c. Calibration curve of TiO_2_ nanoparticles

Figure. S8 a. A sample coated with EPD dried, b. A sample after 24 h of incubation in phosphate buffered saline PBS

Total pages: 6 including title page.

Total Figures: 8

Section S1. Evidence of cell morphology of DPMSCs and Fibroblasts

1. (b)


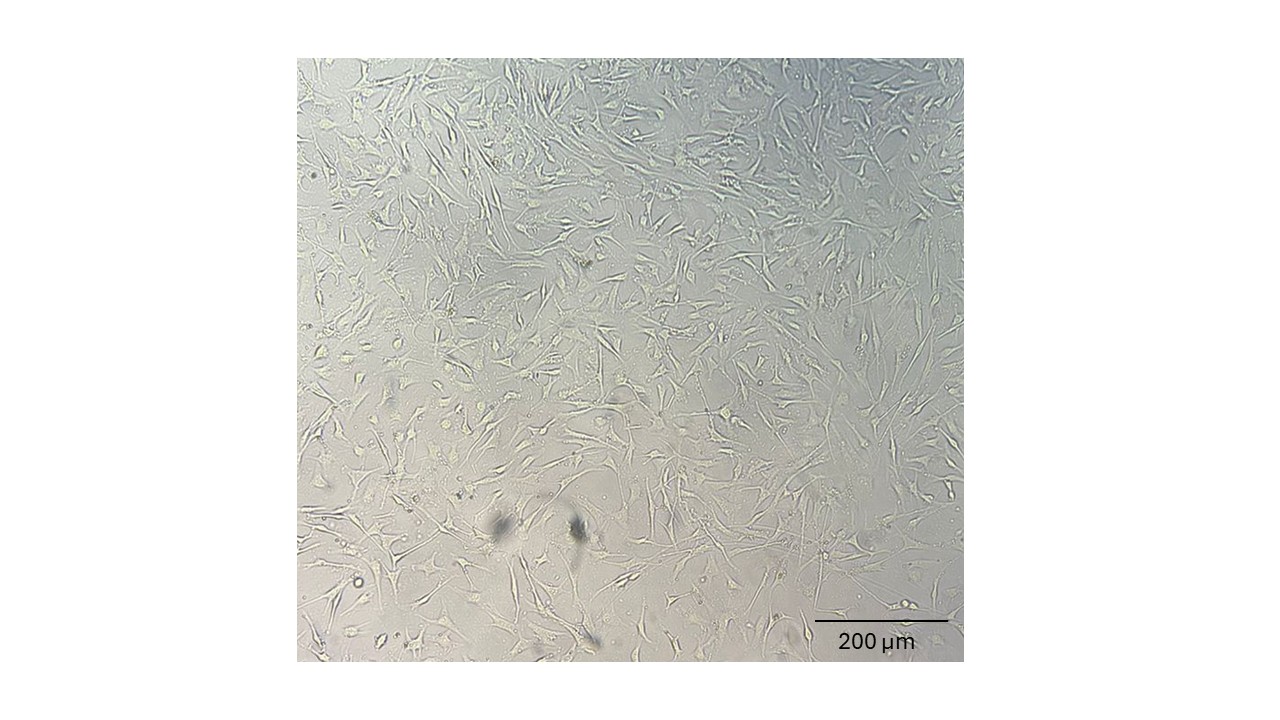

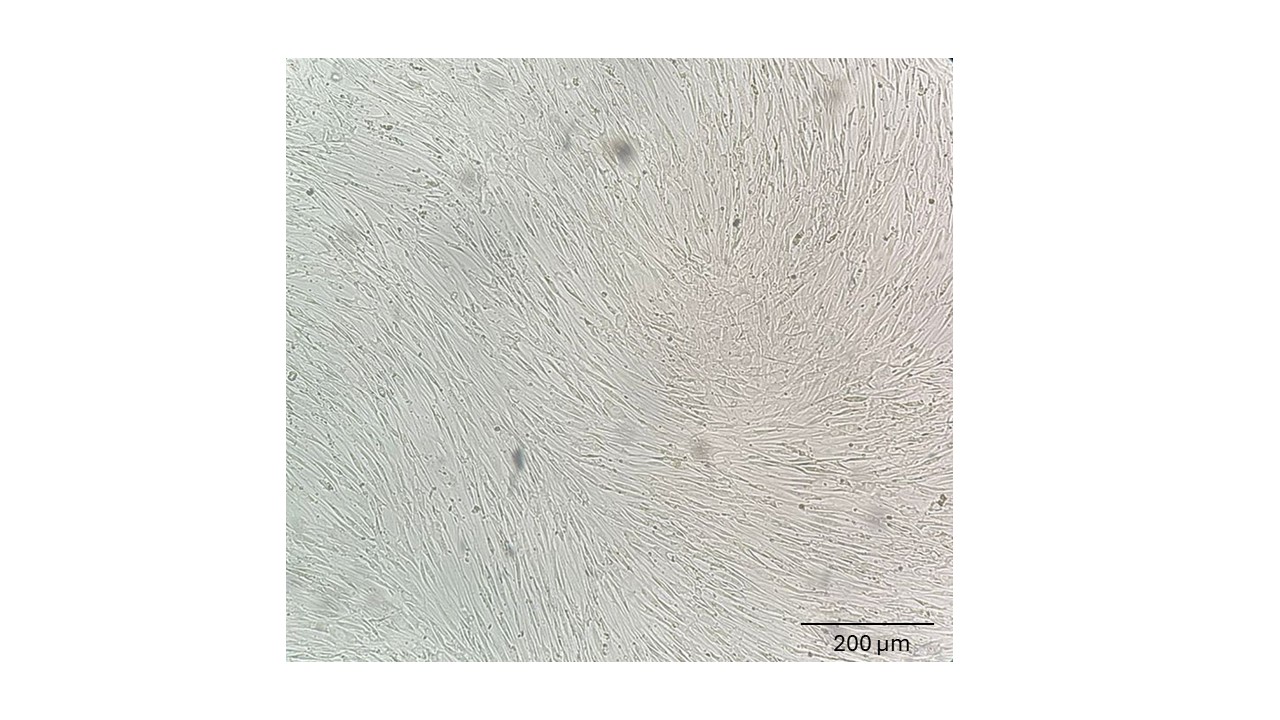


Figure S1. Morphological progression of DPMSCs**.** a. Early passages (P1) showed heterogeneous elongation b. Later passages (P4) achieved higher confluence and organized fibroblast-like patterns, confirming sustained biocompatibility.

Qualitative microscopic observations revealed that the cells were firmly adhered to the substrate, exhibiting a characteristic elongated morphology. The presence of evident cytoplasmic extensions and the transition from an initially spherical shape to a spread morphology indicate successful cellular attachment and spreading. Overall, the cells displayed a typical fibroblast-like appearance consistent with mesenchymal cells in culture, showing no morphological hallmarks of apoptosis or severe cellular stress

Section S2. Fibroblasts cytocompatibility of SPU+NP_G coatings over 3 days

(a) (b) (c)


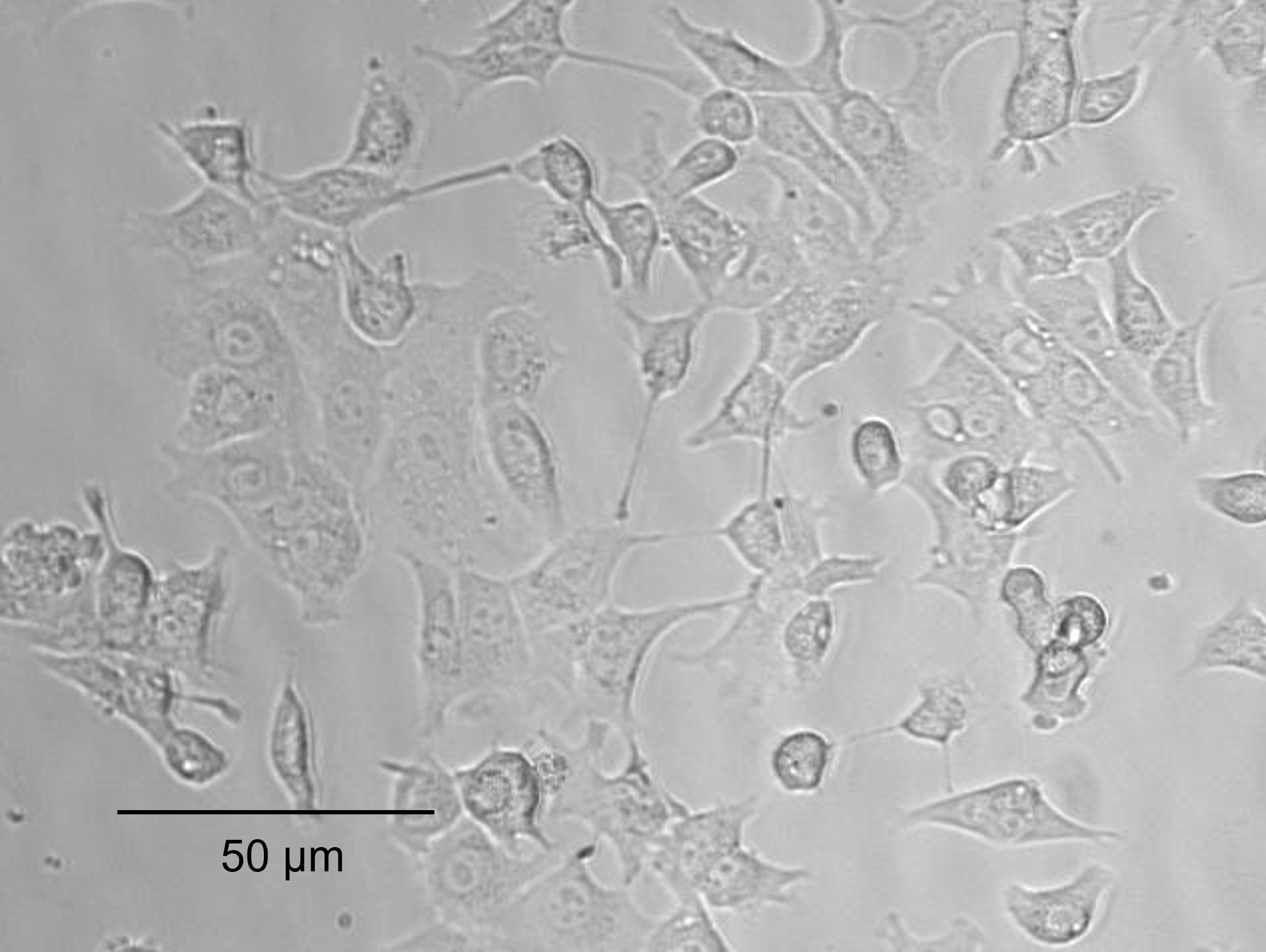

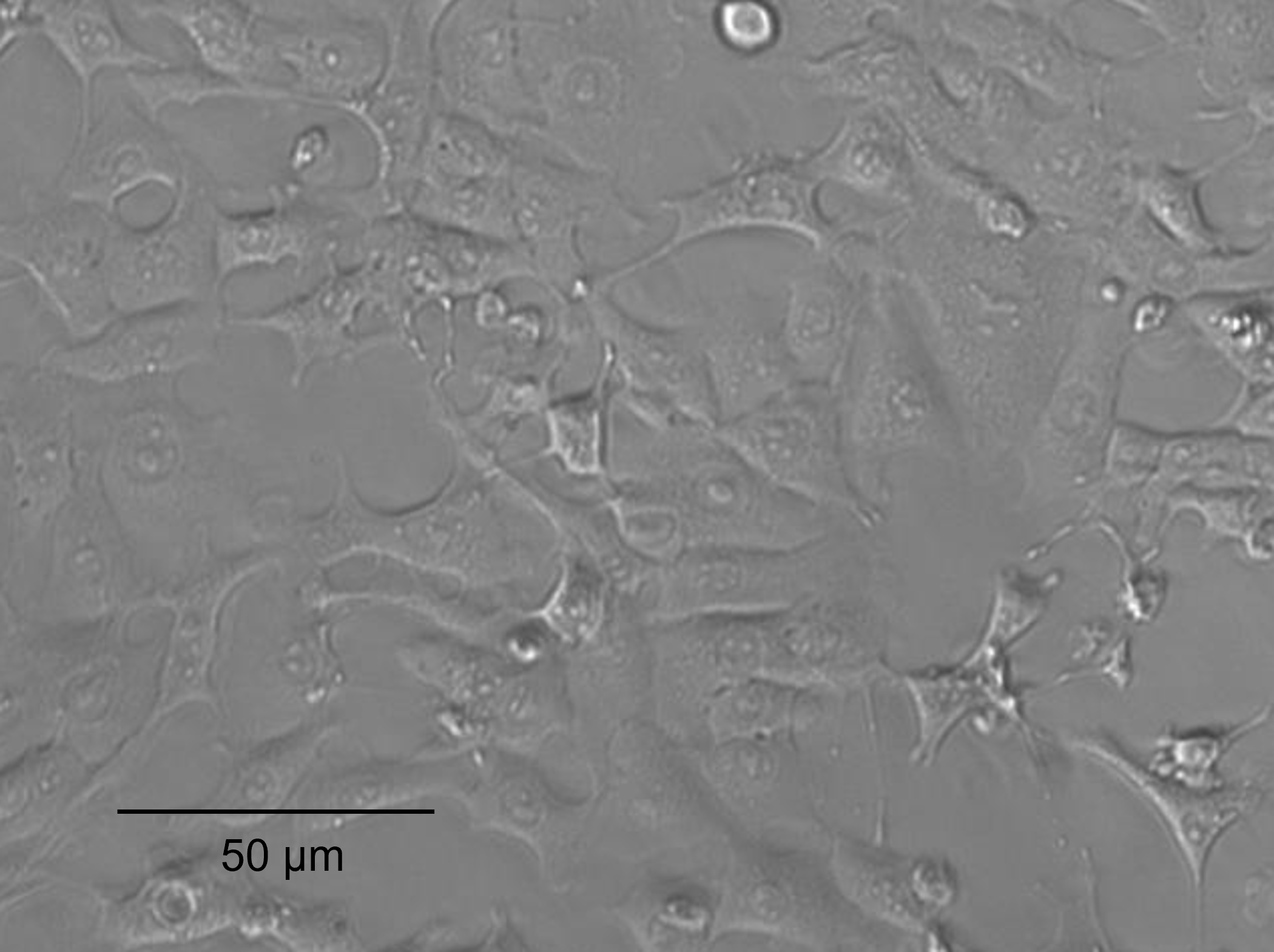

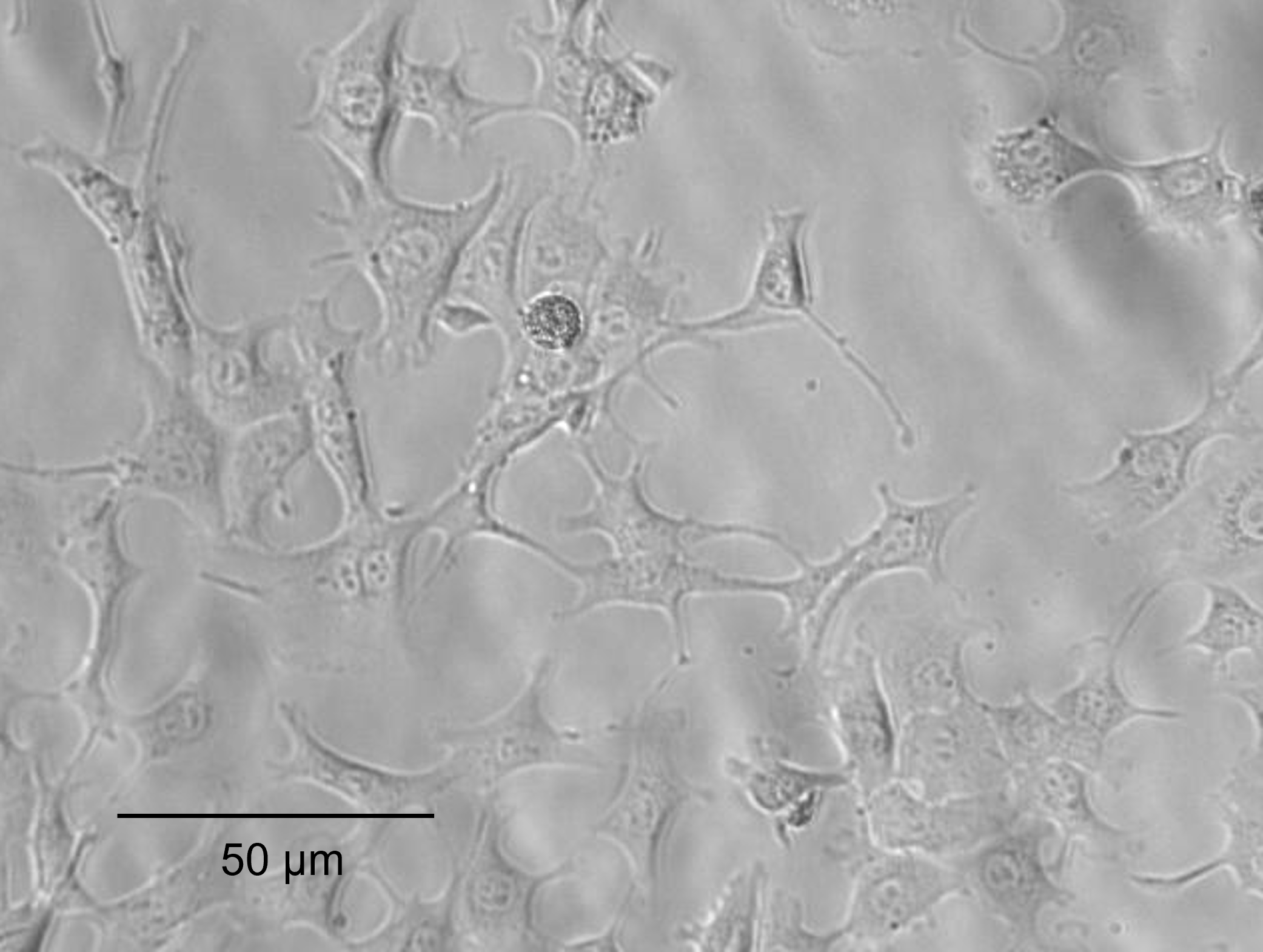


Figure S2. Fibroblasts cytocompatibility of SPU+NP_G coatings over 3 days. (a) Fibroblast viability (ISO 10993-5) showing a transition from non-cytotoxicity (Days 1–2) to a marked proliferative response by Day 3 (>150%). (b–d) Morphological evolution at Days 1, 2, and 3, respectively.

The cytocompatibility of the electrodeposited segmented polyurethane system, incorporating titanium nanoparticles and gentamicin (SPU+NP_G), was confirmed through extract-based assays showing viability levels around 80% on 24h and 48h (Fig. S6a and S6b respectively). At 72 h ((Fig. S6c)), a significant proliferative response was observed, with viability reaching approximately 150%, a bioactive effect likely driven by the stimulatory presence of titanium nanoparticles and the stability of the polyurethane matrix. Qualitative morphological analysis corroborated these quantitative findings, revealing healthy, elongated fibroblasts with extensive cytoplasmic extensions and a lack of cytotoxic stress markers.

Section S3. Cross-section and Pore size of Ti coated with SPU

a.


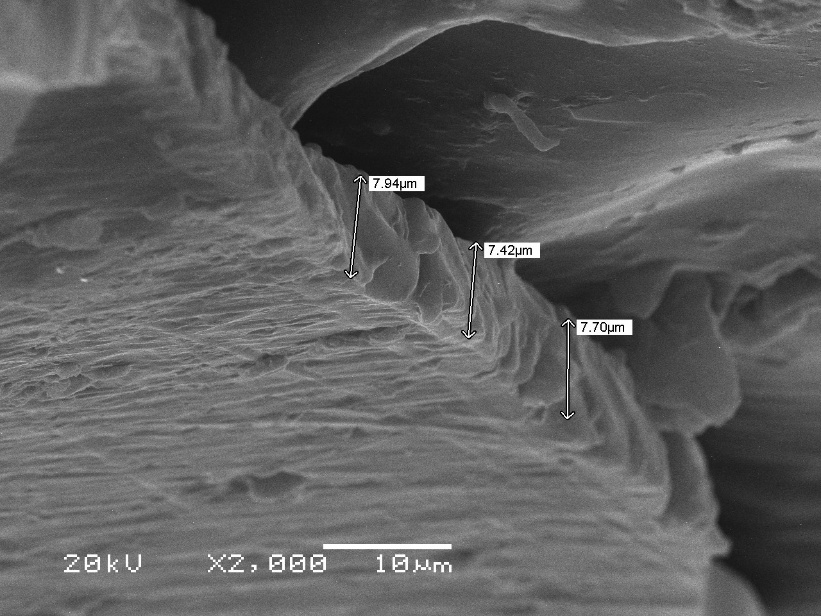


b.


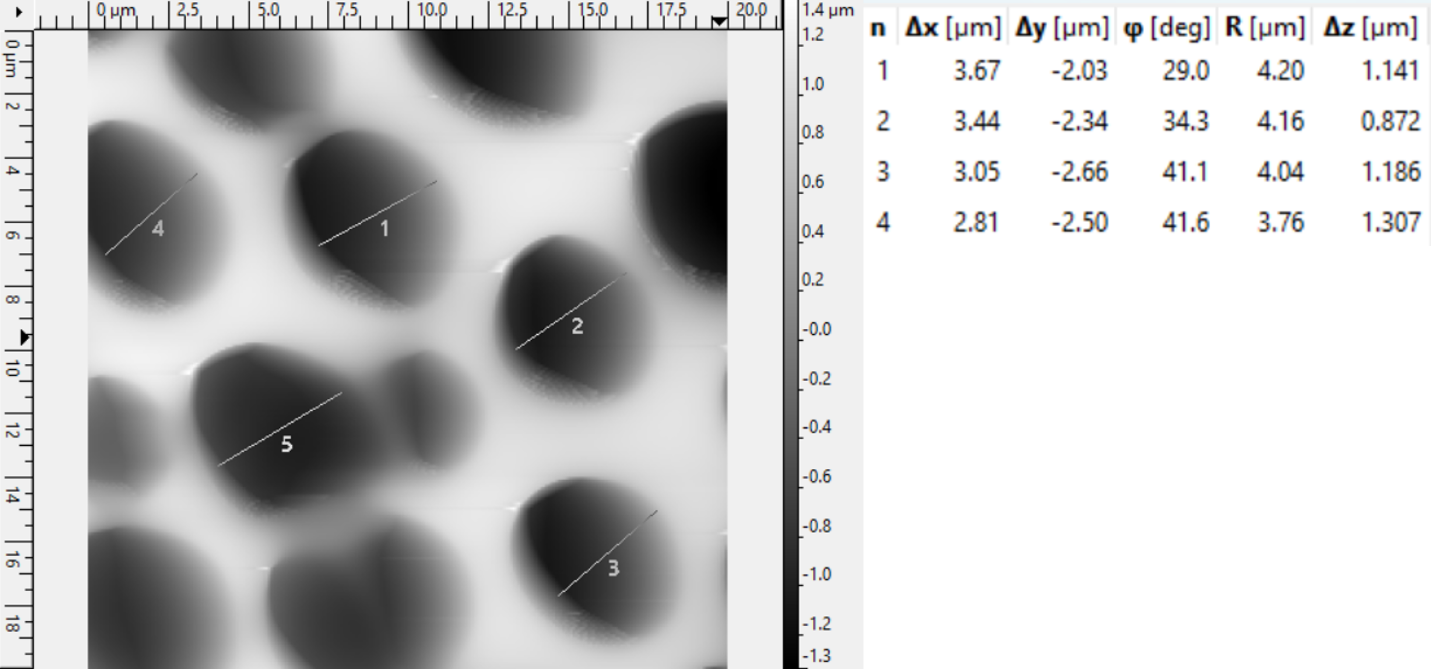


Figure S3. a. SEM image of a cross section of the SPU layer. b. Pore size of a sample of Ti coated with SPU The sample shows an average pore size of 4.12±0.22 μm.

Section S4. Open circuit potential

The electrochemical measurements were conducted using a Gamry PCI4-300 Potentiostat/Galvanostat (Gamry Instruments, Inc., USA) in a conventional three-electrode cell setup. A platinum electrode sourced from StonyLab, USA, served as the auxiliary electrode, while an Ag/AgCl electrode was used as the reference electrode (all potentials mentioned henceforth are in reference to Ag/AgCl) as previously described in [1]. Working electrodes comprised Ti grade 2 and SPU coated Ti coupons, each with a diameter of 14 mm and a surface area of 1.54 cm^2^. Electrolyte comprised an aqueous solution containing 3.5% NaCl by weight. Prior to each experiment, the exposed electrode area underwent polishing using 600 grit SiC abrasive paper, followed by cleaning with ethanol and rinsing with deionized water. To stabilize the surface at the open circuit potential (EOCP) for each sample, specimens were immersed in the electrolyte solution for 1 hour before measurement until stabilization see figure S1.



 a.


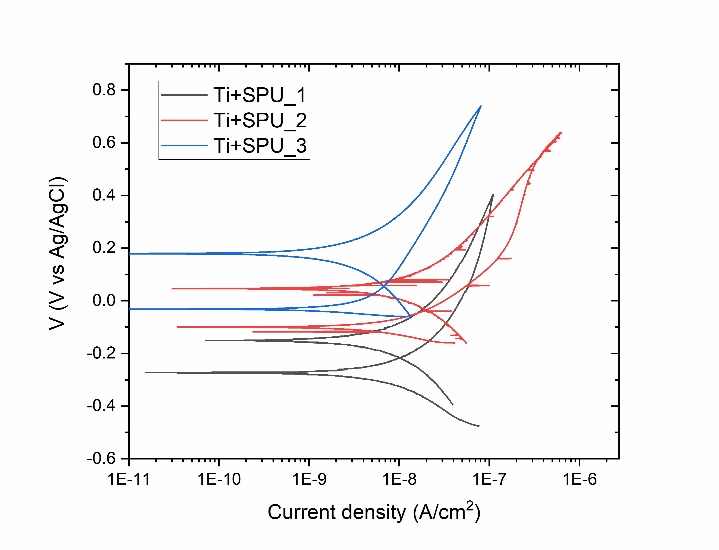

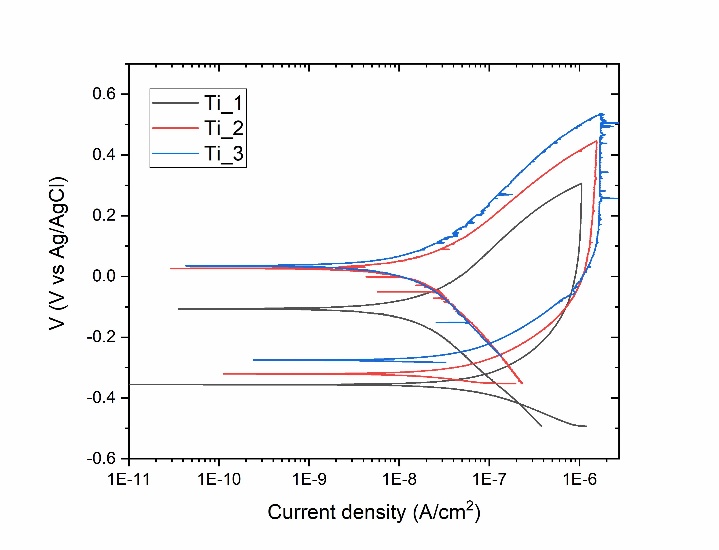
b. c.

Figure S4. a. Measurements of OCP open circuit potential of Ti and Ti+SPU samples. Potentiodynamic polarization cyclic curves in 3.5% NaCl for b. bare Ti plates and c. Ti SPU-coated plates. The coated samples demonstrate a shift toward more noble potentials and reduced current densities

S5. TGA calculations for surface coating density estimation.

Consider a composite coating composed of segmented polyurethane (SPU), gentamicin (GEN), and titanium dioxide NP (NP), with initial masses m_SPU_, m_G_, m_NP_. The TGA-based mass balance is given by:

m_SPU_+ m_G_+m_NP_= m_0_ (1)

*α*_SPU_m_SPU_+*α*_NP_m_NP_+*α*_G_m_G_=Δm (2)

m_NP_=km_SPU_ (3)

Where, m_0_ is the total initial mass of the coating, Δm is the total mass loss measured by TGA, *α*_SPU_+*α*_NP_ and *α*_G_ are the fractional mass losses of SPU, gentamicin, and TiO₂ over the analyzed temperature range, $k$is a known mass ratio between TiO₂ and SPU obtained from the formulation of SPU+NP samples

Table. S5 Parameters measured from TGA

| Component | Fractional mass loss α | Initial mass m (mg) |
| --- | --- | --- |
| NP | 0.94 | 0.0789 |
| G | 0.75 | 0.1686 |
| SPU | 1.00 | 2.2275 |
| Total | — | 2.475 |
| Total mass loss | — | 2.25225 |
|  |  |  |

S6. Agar diffusion antibacterial test

1. b)


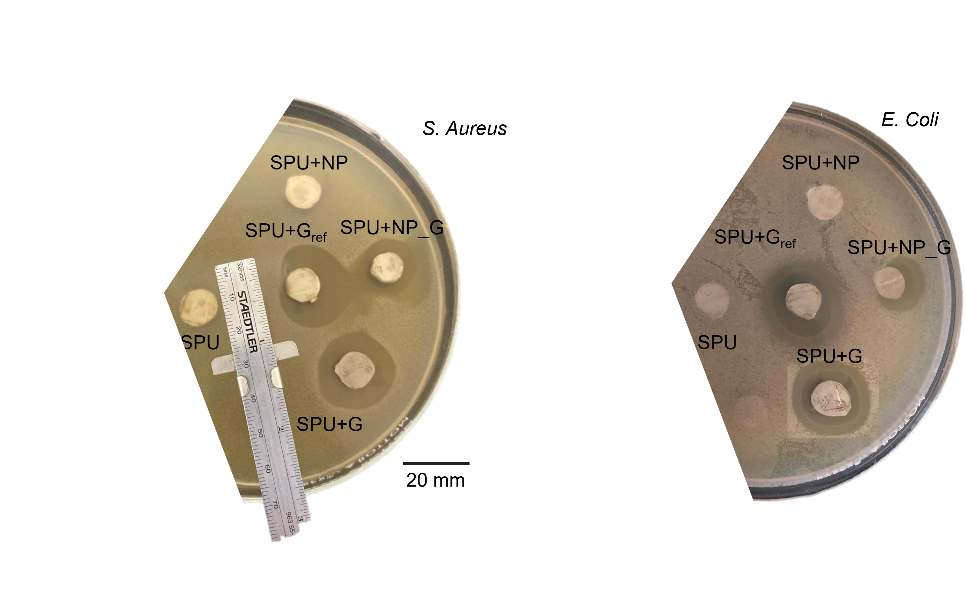


Figure S6. Antibacterial activity of Ti-coated disks evaluated by an agar diffusion (zone of inhibition) assay against (a) *Staphylococcus aureu*s and (b) *Escherichia coli*. Representative images show Ti-coated disks placed on inoculated agar plates after incubation, with the presence of inhibition zones indicating antibacterial activity.

Section S7. Gentamicin calibration curve and TiO2 calibration curve


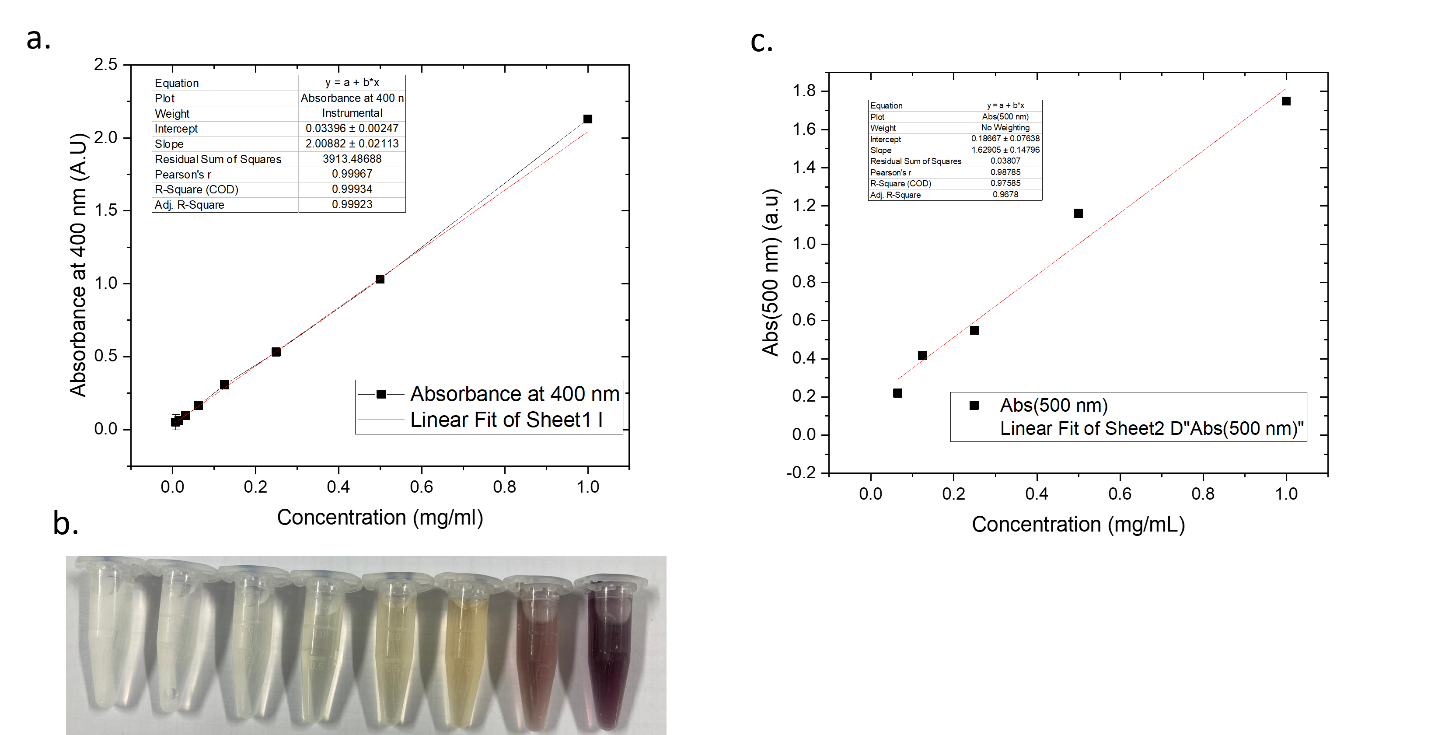


Figure S7. a,b calibration curve of gentamicin by the ninhydrin colorimetric assay, complexes with ninhydrin produce purple color depending on the gentamicin concentration. c. Calibration curve of TiO_2_ nanoparticles

Section S8. TiO_2_ Nanoparticle release


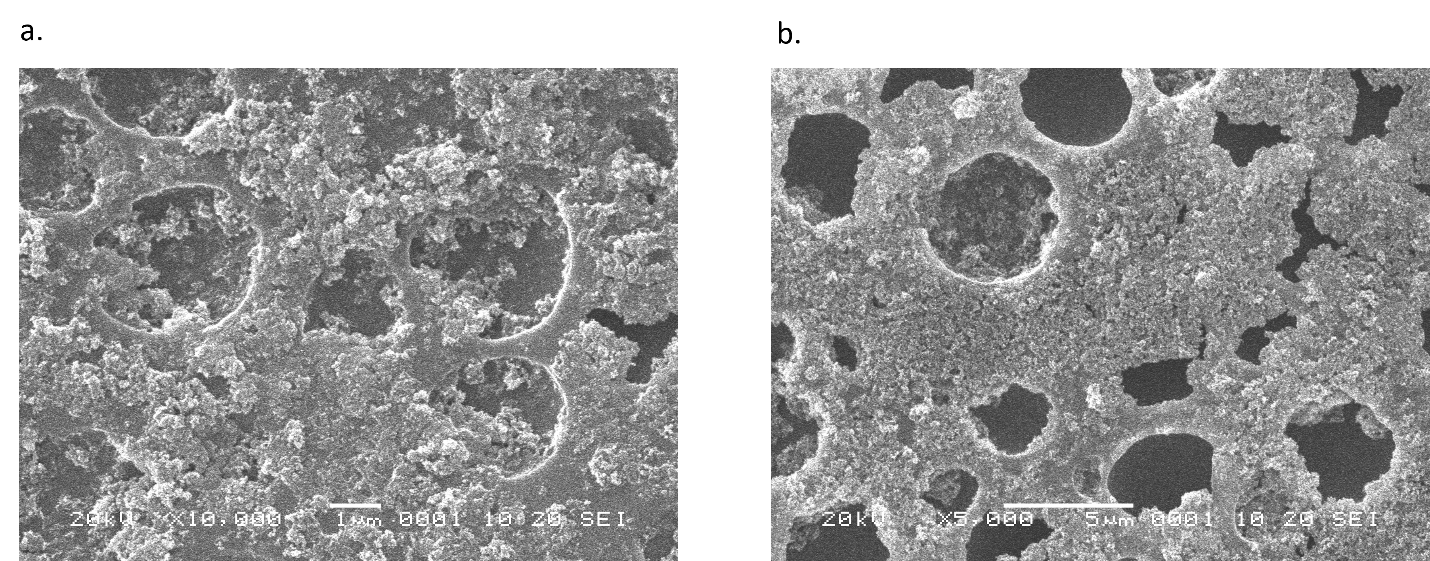


Figure. S8 a. A sample coated with EPD dried, b. A sample after 24 h of incubation in PBS
